# Intranasal Mosaic H1N1 Live Attenuated Influenza Vaccine Elicits Broad Cross‐Reactive Immunity and Protection Against Group 1 and 2 Influenza A Viruses

**DOI:** 10.1002/mco2.70557

**Published:** 2025-12-15

**Authors:** Ximeng Ma, Qi Chen, Yukun Cai, Chen Chen, Jian Lu, Zhuolin Yang, Xue Han, Liangliang Wang, Xuejie Liu, Yuhang Shi, Yuhang Zhang, Li Xin, Yihao Chen, Run Ma, Wantong Pang, Tian Bai, Yuelong Shu

**Affiliations:** ^1^ Key Laboratory of Pathogen Infection Prevention and Control (Peking Union Medical College Ministry of Education) State Key Laboratory of Respiratory Health and Multimorbidity National Institute of Pathogen Biology of Chinese Academy of Medical Science (CAMS)/Peking Union Medical College (PUMC) Beijing China; ^2^ Plastic Surgery Hospital Chinese Academy of Medical Sciences and Peking Union Medical College Beijing China; ^3^ National Institute for Viral Disease Control and Prevention Collaborative Innovation Center For Diagnosis and Treatment of Infectious Diseases Beijing China; ^4^ School of Public Health (Shenzhen) Shenzhen Campus of Sun Yat‐Sen University Shenzhen China; ^5^ Division of HIV/AIDS and Sex‐transmitted Virus Vaccines Institute For Biological Product Control National Institutes for Food and Drug Control (NIFDC) Beijing China; ^6^ Section of Infectious Diseases Department of Internal Medicine Yale University School of Medicine New Haven Connecticut USA

**Keywords:** broad‐spectrum vaccine, influenza, live attenuated influenza vaccine, mosaic, mucosal

## Abstract

Influenza, a highly pathogenic infectious disease, causes nearly half a million deaths annually worldwide. Thus, effective vaccine‐based prevention and control are crucial. Although live attenuated influenza vaccines (LAIVs) can induce mucosal immunity, existing vaccines effectiveness remains relatively low, posing a significant threat to public health. Thus, we developed a novel mosaic H1N1 LAIV candidate by integrating mosaic antigen design with established LAIV technology. This vaccine incorporates most potential T‐cell epitopes of hemagglutinin and neuraminidase antigens into an attenuated master donor strain, ensuring safety and broad immunity. We compared it with commercial monovalent attenuated and inactivated vaccines in mice. The mosaic H1N1 LAIV induced robust cross‐reactive humoral and mucosal immune responses, enhanced antigen‐specific cellular immunity, and established tissue‐resident memory T and B cells in the respiratory tract. Challenge experiments confirmed its protective efficacy against homologous and heterologous strains. It provided complete protection against homologous strains with low epitope similarity and partial protection against the ancestral H3N2 virus. Our study highlights the mosaic H1N1 LAIV as an excellent universal vaccine candidate capable of inducing broad cross‐reactive immune responses and providing robust protection against distinct influenza A viruses, demonstrating a promising strategy to address the limitations of current commercial vaccines.

## Graphical abstract

Our study introduces the mosaic H1N1 LAIV, an intranasal broad‐spectrum influenza vaccine that integrates the mosaic antigen design with LAIV technology. Intranasal boosting in mice elicits robust mucosal, humoral, and cellular immunity, as well as provides extensive protection against both homologous and heterologous influenza strains. Graphical illustrations were generated with Biorender.com.

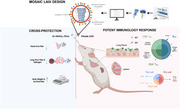

## 1 Introduction

Influenza is an acute respiratory infectious disease caused by influenza viruses, which are classified into four types, A, B, C, and D. Among them, type A viruses (particularly the H1N1 and H3N2 subtypes) and type B viruses are responsible for seasonal epidemics in humans. The pandemic potential of type A viruses stems from their ability to undergo antigenic variations through the surface glycoproteins hemagglutinin (HA) and neuraminidase (NA). Of particular concern is the H1N1 subtype, whose ability to trigger both seasonal epidemics and global pandemics has been repeatedly demonstrated throughout history. The catastrophic 1918 H1N1 pandemic resulted in more than 50 million deaths, and the most recent 2009 H1N1 pandemic caused approximately 500,000 deaths [1]. Seasonal influenza epidemics cause 3–5 million cases of severe disease and up to 500,000 deaths globally each year. According to data from the World Health Organization (WHO) [2], as of January 19, 2025, influenza activity was high and continues to increase in most European and Asian countries (particularly the A(H1N1)pdm09 subtype), with the proportion of patient samples testing positive for influenza exceeding 30% [3].

Vaccination is the most effective measure for influenza prevention and control [4]. Compared with other licensed inactivated vaccines, live attenuated influenza vaccines (LAIVs) offer unique advantages, such as the ability to induce local mucosal immune responses by stimulating secretory immunoglobulin A and tissue‐resident memory T (T_RM_) cells in the respiratory tract. These responses not only prevent infection but also interrupt the transmission of respiratory pathogens, leading to broader, stronger, and longer‐lasting immunity [5, 6, 7]. Furthermore, owing to its noninvasive administration and ease of use, the United States Food and Drug Administration recently approved the LAIV nasal spray FluMist as the first influenza vaccine for self‐ or caregiver administration [8]. However, owing to antigenic drift, all licensed vaccines, including LAIVs provide suboptimal protection (ranging from 10 to 60%), require annual updates, and offer limited protection against emerging pandemic strains [9]. To address these limitations, candidate broad‐spectrum vaccines, such as protein vaccines [10, 11], chimeric HA‐based adjuvant inactivated influenza vaccines (IIVs) [12, 13], and mRNA vaccines [14], have entered clinical trials to improve vaccine efficacy.

T‐cell immunity‐based broad‐spectrum vaccines have been proven to be a central factor in the development of cross‐protective vaccines [15, 16, 17]. Mosaic design employs genetic algorithms to generate a mosaic sequence from a large number of natural sequences, which includes potential T‐cell epitopes with maximal diversity derived from the natural sequences. This approach offers advantages such as broader antigenic coverage and extensive immune responses, and has been applied in the design of highly variable viruses such as HIV and dengue virus [18, 19]. Our team previously developed the computationally optimized mosaic antigens H1m and N1m, which were algorithmically designed to maximize T‐cell epitope coverage on the basis of the H1N1 HA and NA antigens. This antigen design strategy has demonstrated broad‐spectrum protection across multiple platforms, including virus‐like particle, DNA, and protein vaccines, administered intramuscularly and subcutaneously [20, 21, 22, 23].

Here, we developed a mosaic H1N1 LAIV—an intranasal influenza vaccine with broad cross‐reactive immunity and protection against diverse influenza A viruses. Using a reverse genetics system, we integrated the optimized full‐length mosaic HA and NA genes with the six internal genes of the cold‐adapted (ca) and temperature‐sensitive (ts) A/Leningrad/134/17/57(H2N2) (Len/17) master donor virus to construct the mosaic H1N1 LAIV. In vitro studies confirmed the attenuation and stability of the vaccine, supporting its safety profile. Through in vivo mouse experiments, we evaluated the vaccine's safety, immunogenicity, and protective efficacy against both homologous and heterologous strains. In vitro studies confirmed the attenuation and stability of the vaccine, supporting its safety profile. Our study provides a new strategy for the development of universal influenza vaccines to address the limitations of current vaccines in the future.

## 2 Results

### 2.1 Development of a Mosaic H1N1 LAIV as a Universal Vaccine Candidate

**FIGURE 1:**
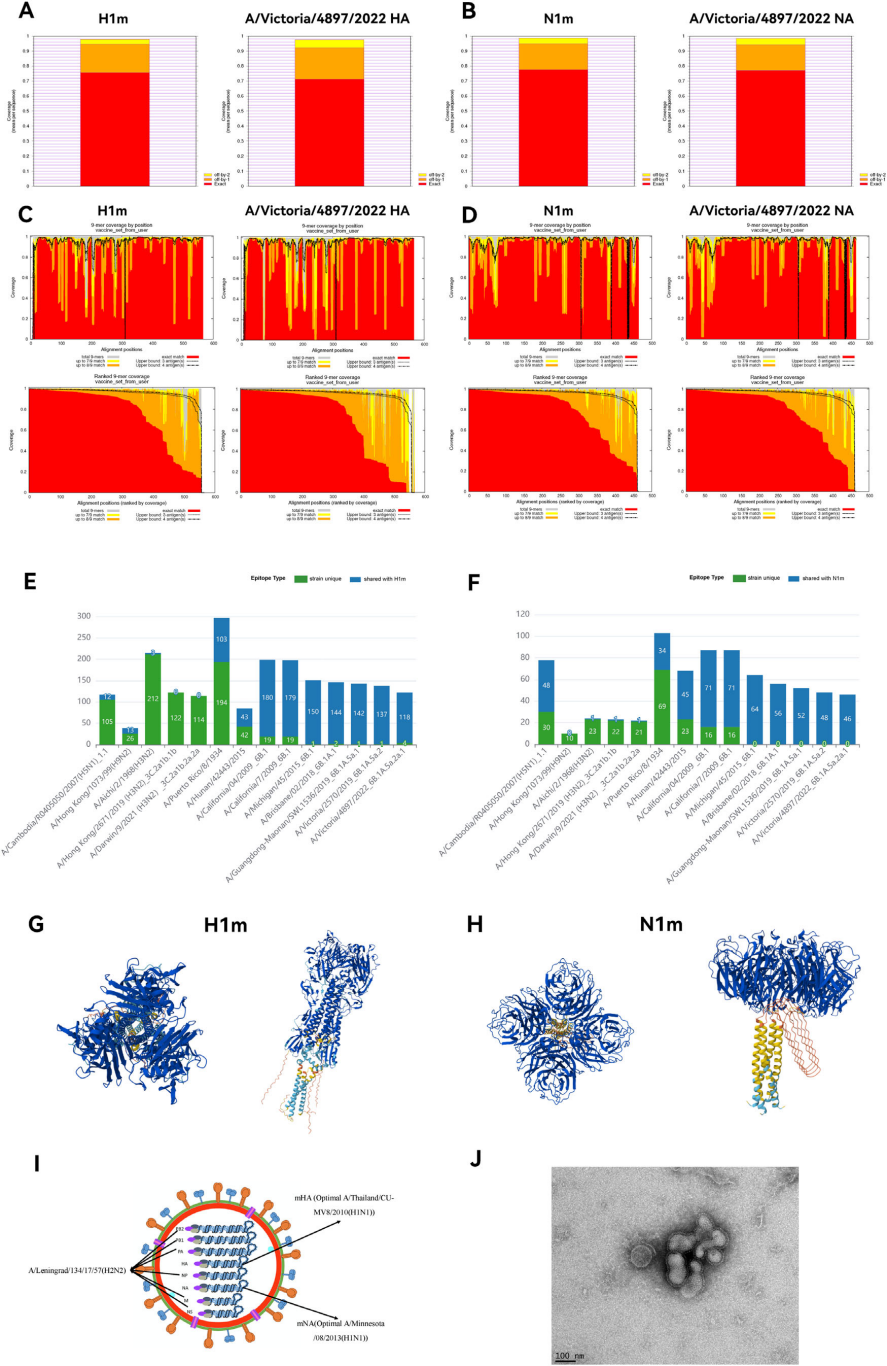
Design and characterization of the mosaic H1N1 LAIV. (A) Comparison of epitope coverage between 09 and 22H1m/N1m and the HA/NA of the latest commercial influenza vaccine strain, A/Victoria/4897/2022 (Vic22). (B) Sequence coverage maps, initially arranged in the natural order of amino acids (top), then reorganized in descending order on the basis of the degree of matching (bottom). (C) Epitope comparison of 09–22H1m and the HA sequences of the influenza A virus strains included in the experiments. (D) Epitope comparison of 09–22N1m and the NA sequences of the influenza A virus strains included in the experiments. (E) Epitope comparison of 09–22H1m and all the HA sequences of the experimental strains. (F) Epitope comparison of 09–22N1m and all the NA sequences of the experimental strains. (G and H) Predicted three‐dimensional structures of the trimer of 09–22H1m (G) and the tetramer of 09–22N1m (H). Different colors indicate different prediction confidence levels. Dark blue indicates very high confidence (pLDDT > 90), light blue indicates high confidence (90 > pLDDT > 70), yellow indicates medium confidence (70 > pLDDT > 50), and orange indicates low confidence (pLDDT < 50). pLDDT, predicted local distance difference test. (I) Schematic representation of the recombinant mosaic H1N1 LAIV containing mH1 and mN1. mH1 is the optimized HA sequence from A/Thailand/CU‐MV8/2010 (GenBank: ADK26546.1), and mN1 is the optimized NA sequence from A/Minnesota/08/2013 (GenBank: AIM39399.1). These sequences are closely similar to those of natural virus strains for better mimicry. The other six internal gene segments are from Len/17. (J) TEM image of the mosaic H1N1 LAIV showing the natural virion morphology (scale bar: 100 nm). HA, hemagglutinin; NA, neuraminidase; TEM, transmission electron microscopy.

To develop a mosaic H1N1 LAIV with broad protective potential against various strains, we first conducted a comprehensive evaluation of our computationally designed 09–22H1m/N1m through three complementary analyses: (i) 9‐mer peptide matching to assess conserved epitope coverage (a 9‐mer peptide refers to a 9‐amino acid peptide sequence within a viral protein (e.g., HA) that can be recognized by the immune system), (ii) epitope continuity analysis to evaluate sequence coverage breadth; and (iii) IEDB epitope alignment to quantify conservation breadth. Our analysis revealed that the epitope coverage of the mosaic antigens was superior to that of the reference strain. A/Victoria/4897/2022 (H1N1, Vic22), the latest WHO‐recommended vaccine strain, served as the reference antigen and the primary comparator in subsequent experiments. With respect to HA, 09–22H1m had higher 9‐mer peptide matching rates (75.62, 94.56, and 97.73% for 9/9, 8/9, and 7/9 matches, respectively) than Vic22 H1 did (71.2, 92.24, and 97.58%, respectively) (Figure 1A). Similarly, for NA, 09–22N1m achieved higher matching rates (77.55, 94.88, and 98.66%, respectively) than Vic22 N1 did (76.96, 94.08, and 98.43%, respectively) (Figure 1B). Epitope continuity analysis confirmed more continuous and extensive sequence coverage for both mosaic antigens (Figure 1C,D). Notably, alignment with the IEDB database (1236 HA and 272 NA epitopes) revealed that 09–22H1m matched 183 epitopes, representing a 47% increase compared with the 124 epitopes matched by Vic22 H1. Similarly, N1m matched 74 epitopes, a 61% increase compared with the 46 epitopes matched by Vic22 N1. We further expanded the comparison to a broader range of strains. HA epitope comparison revealed that 09–22H1m shares the most epitopes with H1N1 strains, particularly post‐2009 variants, indicating near‐complete epitope conservation. Notably, 09–22H1m exhibited partial epitope sharing with the H9N2 and H5N1 strains (Figures 1E and S1). Furthermore, NA epitope comparison revealed that 09–22N1m shared the most epitopes with the H1N1 and H5N1 strains, particularly with post‐2009 H1N1 strains, with which the epitopes were almost entirely shared (Figures 1F and S1). Some epitope overlap was observed between the 09–22H1m/N1m and H3N2 strains, in which 3 HA/1 NA epitopes were shared with the A/Aichi/2/1968 (X31, H3N2) strain. Collectively, these findings demonstrate the broad T‐cell epitope coverage of 09–22H1m and 09–22N1m, supporting their potential as cross‐reactive protective antigens against multiple influenza A virus strains.

Using AlphaFold3, we predicted the three‐dimensional structures of 09–22H1m and 09–22N1m. The predicted models suggested that both proteins could form trimeric (09–22H1m) and tetrameric (09–22N1m) structures (Figure 1G,H). These structural predictions are consistent with the known oligomeric states of HA and NA proteins, which are critical for maintaining antigenic integrity and functional efficacy during vaccine development.

To optimize immunogenicity and expression, we designed and synthesized the mosaic nucleotide sequences mH1 and mN1 (Figure 1I and Supplementary Data 1). By using Len/17 as the attenuated backbone, we successfully rescued the mosaic H1N1 LAIV and the 23–24H1N1 LAIV through reverse genetics technology, with the latter serving as one of the controls and representing a commercial vaccine strain. The second‐passage (P2) egg‐grown viruses presented hemagglutination titers of 128 and a 50% embryo infectious dose (EID_50_) of 7.3 log_10_ EID_50_/mL. Sanger sequencing confirmed the identity of all the viral genes, and the virus was passaged in chicken embryos at 33°C. Notably, the titer of the vaccine candidate reached 8.6 log_10_ EID_50_/mL in the P3 generation and 8.9 log_10_ EID_50_/mL in the P4 generation, indicating robust viral replication. The mosaic H1N1 LAIV exhibited high titers and displayed intact viral particle morphology on transmission electron microscopy (TEM) (Figure 1J).

In summary, 09–22H1m/N1m has wide coverage of T‐cell epitopes, and its predicted structure is intact. The mosaic H1N1 LAIV can be successfully constructed and has an intact viral particle morphology, which provides crucial support for subsequent experimental verification and vaccine development.

### 2.2 Mosaic H1N1 LAIV Exhibits Attenuation and Genetic Stability

On the basis of the relevant literature [24, 25], as well as the temperatures of the upper respiratory tract and lower respiratory tract, we evaluated the ts and ca phenotypes at 29, 33, and 39°C, as well as the growth of the virus in specific‐pathogen‐free (SPF) embryonated chicken eggs. Both the commercial 23–24H1N1 LAIV and the mosaic H1N1 LAIV exhibited efficient replication at 33 and 29°C. Neither virus was able to replicate at 39°C (Table 1). These results demonstrate that the mosaic H1N1 vaccine has ca and ts phenotypes similar to those of the commercially available LAIV.

**TABLE 1:** Results of the ts and ca assays of the mosaic H1N1 LAIV.

	Virus titer [log_10_(EID_50_/mL)]			Phenotype
	29°C	33°C	39°C	
23‐24H1N1 LAIV	5.5	7.5	–^a^	ts, ca
Mosaic H1N1 LAIV	6.5	7.3	–—	ts, ca

^a^ Lower than 2.

We also evaluated the genetic stability of the mosaic H1N1 LAIV. After 10 passages, only one mutation was observed, namely, alanine (A) to aspartic acid (D) at position 187 in the HA sequence. This mutation did not affect the ts or ca phenotype [26]. These data suggest that the vaccine strain exhibited high genetic stability after several replications in embryos.

Overall, the attenuation characteristics of the mosaic H1N1 LAIV are similar to those of commercially available LAIVs. The mosaic LAIV can act effectively in the upper respiratory tract and prevent severe infections. It also exhibits high genetic stability after multiple passages, and the mutation detected did not affect its important characteristics. These results provide a solid foundation for the mosaic LAIV to be a candidate universal LAIV.

### 2.3 Intranasal Mosaic H1N1 LAIV Exhibits Good Safety in Vivo

**FIGURE 2:**
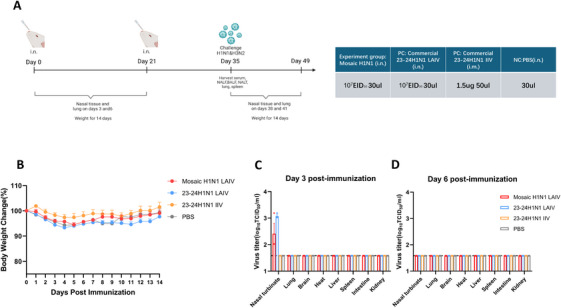
Safety evaluation of the mosaic H1N1 LAIV in BALB/c mice. (A) Experimental design for immunization and sample collection. (B) Body weights over 14 days after immunization (*n* = 5 mice per group). The data are presented as a line chart. (C) Viral titers of NTs, lungs, and other tissues on Day 3 (*n* = 3 mice per group). The data are presented as a bar chart. (D) Viral titers of NTs, lungs, and other tissues on Day 6 (*n* = 3 mice per group). The data are presented as a bar chart with individual data points. The limit of detection was 1.52 log_10_ TCID_50_/mL. BALF, bronchoalveolar lavage fluid; NALF, nasal lavage fluid; NT, nasal tissue.

The immunization strategy involved two doses in female BALB/c mice (6–8 weeks of age) (Figure 2A). All the mice survived for 14 days without significant body weight loss (Figure 2B). No virus was detected in the lungs, brains, hearts, livers, spleens, intestines, or kidneys on Day 3 or 6 (Figure 2C,D). On Day 3, slight virus replication was observed in nasal turbinates (NTs), with titers of approximately 2.4 log_10_ TCID_50_/mL in the mosaic H1N1 LAIV group and 3.1 log_10_ TCID_50_/mL in the 23–24H1N1 LAIV group (Figure 2C). On Day 6, the virus titer in the NTs of the mosaic H1N1 LAIV group was below the limit of detection (Figure 2D). Histopathological examination revealed that the lung tissue structure of the mice in all the groups was intact, with no significant pathological changes observed in the alveoli, bronchi, or blood vessels (Figure S2). These results confirm that the safety of the mosaic H1N1 LAIV is comparable to that of the commercial vaccine.

### 2.4 Intranasal Mosaic H1N1 LAIV Elicits Broad Cross‐Reactive Mucosal and Systemic Antibody Responses Against Homologous and Heterologous Influenza A Viruses

To assess the breadth of antibodies induced by the mosaic H1N1 LAIV, serum, BALF, and NALF were collected from BALB/c mice 14 days postboost.

**FIGURE 3:**
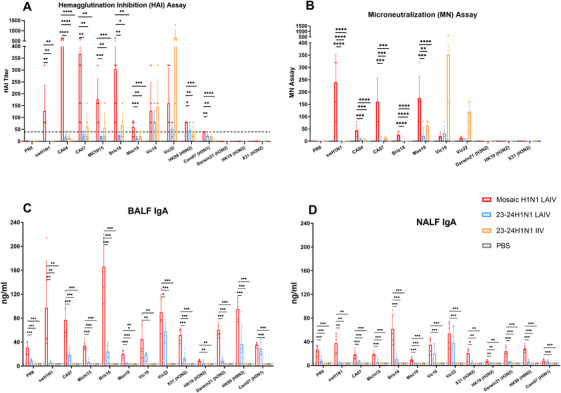
The mosaic H1N1 LAIV elicits cross‐reactive antibody responses against homologous and heterologous influenza strains. (A) A HAI assay was conducted to assess cross‐reactive HAI antibody responses in mice on Day 35. The target antigens included representative H1N1 strains (the ancestral strain PR8, the swine influenza virus swH1N1, pdm09, and the WHO‐recommended seasonal H1N1 vaccine strains from 2009 to 2025, namely, CA07, Mich15, Bris18, Mao19, Vic19, and Vic22) and heterologous viruses (H5N1: Cam07; H9N2: HK99; and H3N2: X31, HK19, and Darwin21) (*n* = 5 mice per group). (B) An MN assay was carried out to measure neutralizing antibody responses to representative H1N1 strains (PR8, swH1N1, and the WHO‐recommended seasonal H1N1 vaccine strains CA07, Bris18, Mao19, Vic19, and Vic22) in mice on Day 35 (*n* = 5 mice per group). (C and D) Antigen‐specific IgA levels in the BALF (C) and NALF (D) were measured on Day 35 in mice by ELISA. The target antigens included H1N1 strains (PR8, swH1N1, and WHO‐recommended seasonal H1N1 vaccine strains from 2009 to 2025) and heterologous viruses (H3N2: X31, HK19, Darwin21; H5N1: Cam07; and H9N2: HK99) (*n* = 5 mice per group). The limit of detection was 3.91 ng/mL. The data are presented as the means ± SDs. Statistical analysis for (C) and (D) was performed via a nonparametric Kruskal–Wallis test or one‐way ANOVA, with significance indicated as *p < 0.05, **p < 0.01, and ***p < 0.001. The data are presented as bar charts with individual data points. BALF, bronchoalveolar lavage fluid; HAI, hemagglutination inhibition; MN, microneutralization; NALF, nasal lavage fluid.

We first evaluated HAI antibodies against representative influenza A strains, including the ancestral strain A/Puerto Rico/8/1934 (PR8), the swine influenza virus A/Hunan/42443/2015 (swH1N1), the 2009 pandemic strain A/California/04/2009 (CA04), WHO‐recommended seasonal H1N1 vaccine strains (2009–2025: A/Victoria/4897/2022 (Vic22), A/Victoria/2570/2019 (Vic19), A/Guangdong‐Maonan/SWL1536/2019 (Mao19), A/Brisbane/02/2018 (Bris18), A/Michigan/45/2015 (Mich15) and A/California/7/2009 (CA07)), and heterologous viruses including A/Aichi/2/1968 (X31, H3N2), A/Hongkong/2671/2019 (HK19, H3N2)A/Hongkong/1073/99 (HK99, H9N2), and A/Cambodia/R0405050/2007 (Cam07, H5N1) (Figure 3A). HAI is regarded as the standard in influenza virus serology, with an HAI titer of ≥40 considered protective in adults [6, 27]. Here, the mosaic H1N1 LAIV elicited broad cross‐reactive antibody responses, which resulted in titers ≥40 against eight out of nine H1N1 strains (88.9%, titers ranging from 40 to 640), including swH1N1 (titers from 80 to 320). Notably, it also induced substantial cross‐HAI reactivity against HK99 (H9N2, titer 80), although it exhibited low reactivity against Cam07 (H5N1, titer 40). In contrast, 23–24H1N1 LAIV control had limited effects and met the protective thresholds against only two recent WHO‐recommended strains and the H9N2 strain. The 23–24H1N1 IIV group exhibited strong responses to only two recent strains and limited HAI antibodies to swH1N1, CA04, Mao19, and Cam07 (H5N1). No HAI antibody was detected against PR8, heterologous H3N2 in any group.

Next, we assessed neutralizing antibodies via the MN assay, which provides enhanced sensitivity and specificity [27, 28] (Figure 3B). Mosaic H1N1 LAIV immunization induced broad cross‐reactive MN responses, with neutralization of seven out of eight H1N1 strains (87.5%). In contrast, the 23–24H1N1 LAIV and IIV elicited neutralizing antibodies against only the three most recent WHO‐recommended strains. The MN antibody pattern aligned with the HAI results, confirming the functional cross‐reactivity of these antibodies. No MN titer was detected against PR8 in any group.

Given that IgA serves as the first line of defense against infection [29], we further investigated a diverse panel of influenza antigen‐specific IgA levels in the BALF (Figure 3C) and NALF (Figure 3D), including PR8, swH1N1, WHO‐recommended seasonal H1N1 vaccine strains (2009–2025), and heterologous influenza virus strains including X31 (H3N2), Darwin21 (H3N2), Cam07 (H5N1), and HK99 (H9N2). The mosaic H1N1 LAIV group exhibited broad cross‐reactivity and significantly higher IgA concentrations against all representative influenza strains. Specifically, compared with those in all the control groups (treated with 23–24H1N1 LAIV, 23–24H1N1 IIV, and phosphate‐buffered saline (PBS)), robust responses were observed against H1N1, strains including PR8 (*p* < 0.001, BALF/NALF), swH1N1 (*p* < 0.01, BALF/NALF), 09–21 WHO‐recommended strains CA07/Mich15/Bris18/Mao19 (all *p* < 0.001, BALF/NALF), and heterologous strains, including X31 (H3N2, *p* < 0.001 in BALF, p<0.05 in NALF), HK19 (H3N2, *p* < 0.01, NALF), Darwin21 (H3N2, *p* < 0.01, BALF/NALF), and HK99 (H9N2, *p* < 0.001, BALF/NALF). Compared with those observed in the 23–24H1N1 IIV and PBS groups, robust responses were observed against the recent WHO‐recommended H1N1 strains Vic19 (*p* < 0.01 in BALF, *p* < 0.001 in NALF) and Vic22 (*p* < 0.001, BALF/NALF), as well as the heterologous strain Cam07 (H5N1, *p* < 0.001, BALF/NALF). While IgA levels in the NALF were comparable to or slightly lower than those in the BALF, the results collectively demonstrated that the mosaic H1N1 LAIV elicited robust mucosal antibody responses in both the upper and lower respiratory tracts, targeting both homologous and heterologous influenza subtypes. In contrast, the 23–24H1N1 LAIV exhibited comparable IgA responses against only Vic19, Vic22, and Cam07 (H5N1), with minimal or no reactivity to other homologous and heterologous strains. Both the 23–24H1N1 IIV and PBS controls presented undetectable IgA levels across all the antigens. Overall, the mosaic H1N1 LAIV elicits broad cross‐reactive systemic and mucosal antibody responses encompassing all tested influenza A subtypes, including ancestral, seasonal, and heterologous strains, demonstrating broader reactivity than conventional vaccine platforms.

### 2.5 Intranasal Mosaic H1N1 LAIV Elicits Antigen‐Specific Cellular Immune Responses Against Homologous and Heterologous Influenza A Viruses

T cells play a critical role in combating respiratory infections and are highly antigen specific [30]. We evaluated the antigen‐specific T‐cell responses elicited by the mosaic H1N1 LAIV. On Day 35, splenic cells were stimulated in vitro with PR8 (H1N1) or X31 (H3N2) antigens, followed by enzyme‐linked Immunospot (ELISpot) analysis of the secretion of IFN‐γ (Th1), IL‐4 (Th2), and IL‐17A (Th17), which are key cytokines that regulate inflammation, antiviral immunity, and viral clearance [30, 31, 32].

**FIGURE 4:**
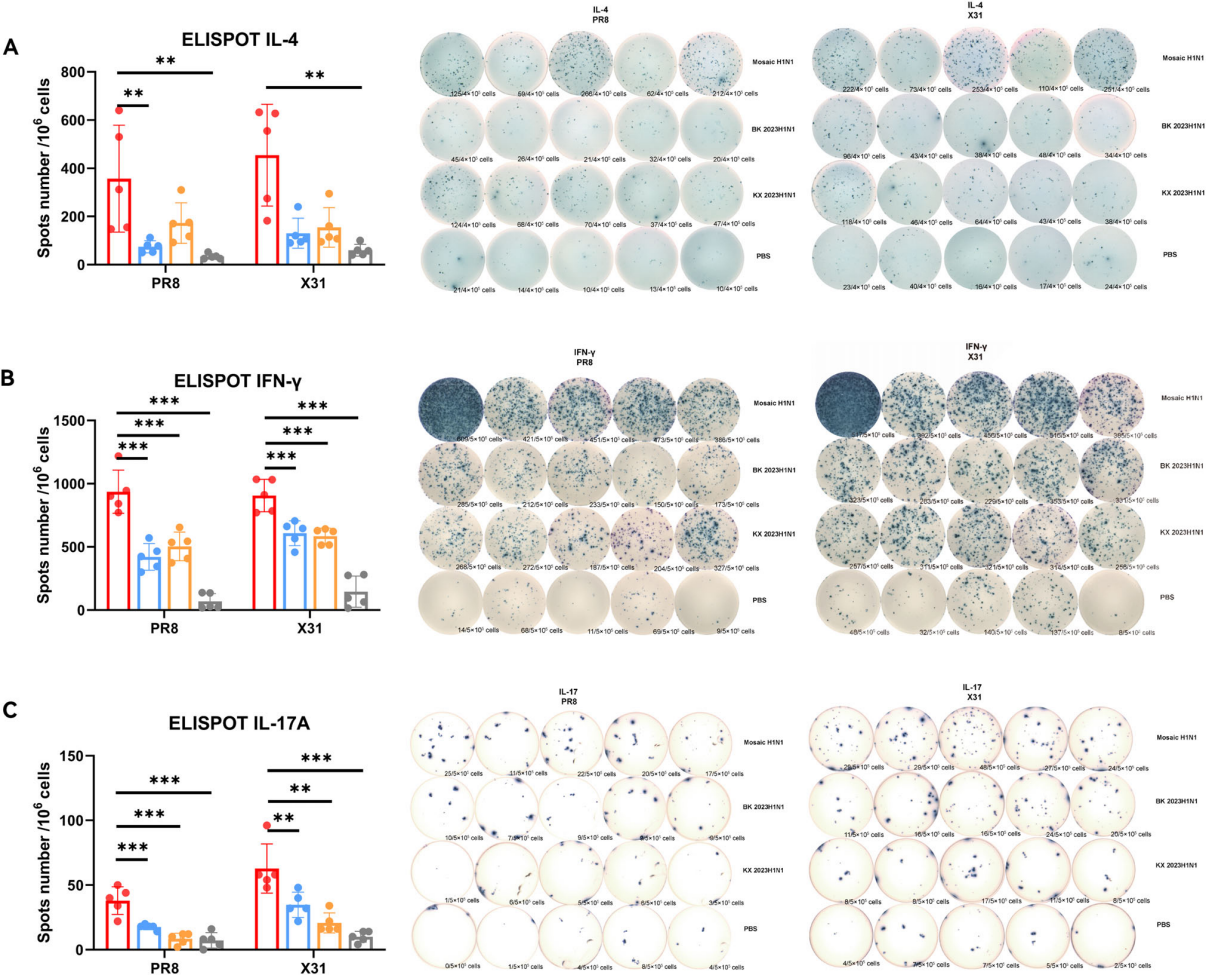
Mosaic H1N1 LAIV induces antigen‐specific cytokine‐producing T cells. Splenocytes were isolated on Day 35 and stimulated with either homologous (PR8 or H1N1) or heterologous (X31 or H3N2) influenza antigens in ELISpot assays to quantify the secretion of (A)IL‐4 (Th2), (B)IFN‐γ (Th1), and (C) IL‐17A (Th17) (*n* = 5 mice per group). The data are presented as the means ± SDs. Statistical analysis was performed via one‐way ANOVA, with significance indicated as *p < 0.05, **p < 0.01, and ***p < 0.001. The data are presented as bar charts with individual data points. IFN‐γ, interferon‐γ; IL‐4, interleukin‐4; IL‐17A, interleukin‐17A.

The mosaic H1N1 LAIV group presented more IL‐4‐secreting cells (all *p* < 0.01 vs. LAIV and PBS) (Figure 4A) and significantly PR8‐specific IFN‐γ‐ and IL‐17A‐secreting cells (all *p* < 0.001) (Figure 4B,C). Surprisingly, X31‐specific responses (IFN‐γ/IL‐4/IL‐17A) were similarly strengthened. The mosaic H1N1 LAIV group had significantly more X31‐specific IFN‐γ‐secreting (all *p* < 0.001), IL‐17A‐secreting (all *p* < 0.01) cells, as well as more IL‐4‐secreting cells (*p* < 0.01 vs. PBS) (Figure 4A–C), demonstrating cross‐reactivity against heterologous H3N2 strains. The 23–24H1N1 LAIV/IIV control groups presented similar results, whereas the PBS group presented negligible responses. These findings suggest that the mosaic H1N1 LAIV uniquely activates Th1, Th2, and Th17 cells, enabling adaptive immune responses against both homologous and heterologous strains.

### 2.6 Flow Cytometry Confirmed the Antigen‐Specific CD8^+^ and CD4^+^ T‐Cell Responses Against Homologous and Heterologous Influenza A Viruses

**FIGURE 5:**
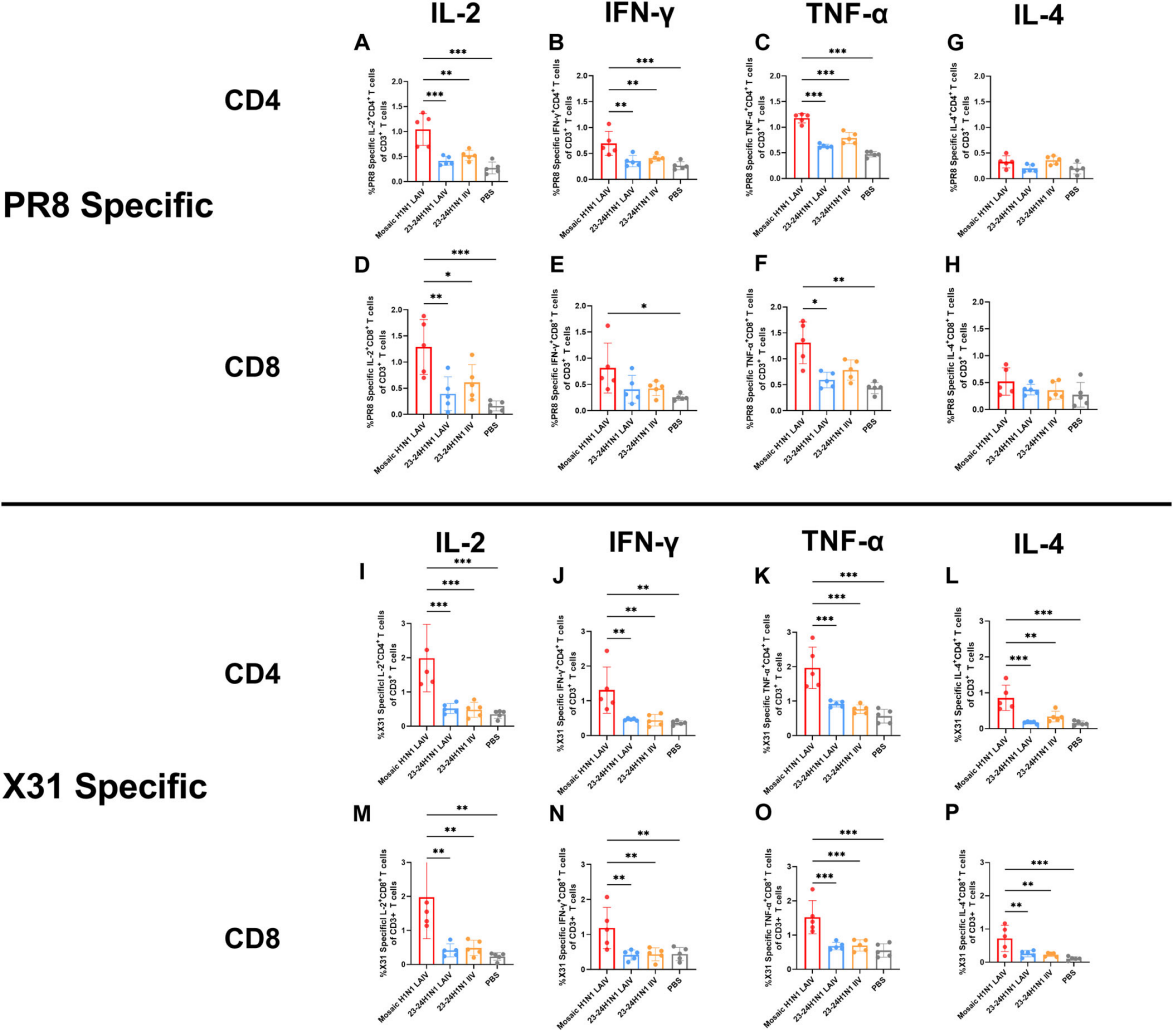
The mosaic H1N1 LAIV elicits broad antigen‐specific T‐cell responses. Splenocytes collected on Day 35 after immunization were stimulated with (A–H) homologous PR8 (H1N1) or (I–P) heterologous X31 (H3N2) influenza antigens and subjected to intracellular cytokine staining and flow cytometry analysis. Frequencies of CD4^+^ IL‐2^+^(A), CD4^+^ IFN‐γ^+^ (B), CD4^+^ TNF‐α^+^(C), CD4^+^ IL‐4^+^ (G), CD8+ IL‐2^+^ (D), CD8^+^ IFN‐γ^+^ (E), CD8^+^ TNF‐α^+^ (F), and CD8^+^ IL‐4^+^ (H) T cells in response to PR8 and CD4^+^ IL‐2^+^ (I), CD4^+^ IFN‐γ^+^ (J), CD4^+^ TNF‐α^+^ (K), CD4^+^ IL‐4^+^ (L), CD8^+^ IL‐2^+^ (M), CD8^+^ IFN‐γ^+^ (N), CD8^+^ TNF‐α^+^ (O), and CD8^+^ IL‐4^+^ (P) T cells in response to X31. The data are presented as the means ± SDs (*n* = 5 mice per group). Statistical analysis was performed via the nonparametric Kruskal‒Wallis test or one‐way ANOVA, with significance indicated as *p < 0.05, **p < 0.01, and ***p < 0.001. The data are presented as bar charts with individual data points. IFN‐γ, interferon‐γ; IL‐4, interleukin‐4; IL‐2, interleukin‐2; TNF‐α, tumor necrosis factor‐α.

Influenza virus‐specific CD4^+^ and CD8^+^ T cells play critical roles in mediating protective immunity [33]. To further investigate antigen‐specific T‐cell responses, we performed flow cytometry analysis of splenocytes following stimulation with influenza antigens. Under stimulation with the low‐homology ancestral PR8 strain, the mosaic H1N1 LAIV elicited significantly greater frequencies of CD4^+^ IL‐2^+^ (all *p* < 0.001), IFN‐γ^+^ (all *p* < 0.01) and TNF‐α^+^ (all *p* < 0.001) cells (Figure 5A–C) and CD8^+^ IL‐2^+^ (*p* < 0.001 vs. LAIV and PBS, *p* < 0.01 vs. IIV) cells (Figure 5D), as well as larger CD8^+^ IFN‐γ^+^ (*p* < 0.05 vs. PBS) and TNF‐α^+^ (*p* < 0.01 vs. PBS) populations (Figure 5E,F). While the number of CD4^+^ and CD8^+^ IL‐4^+^ cells was modestly greater in the mosaic H1N1 LAIV group than in the 23–24H1N1 LAIV and PBS groups, the difference was not statistically significant (Figure 5G,H). Notably, under stimulation with heterologous A/Aichi/2/1968 (X31, H3N2), the mosaic H1N1 LAIV induced significantly greater frequencies of CD4^+^ T cells that produced the cytokines IL‐2 (all *p* < 0.01), IFN‐γ (all *p* < 0.01), TNF‐α (all *p* < 0.001), and IL‐4 (*p* < 0.001 vs. LAIV and PBS, *p* < 0.01 vs. IIV) (Figure 5I–L). Moreover, the mosaic H1N1 LAIV elicited significantly greater frequencies of CD8^+^ T cells that generated the cytokines IFN‐γ (all *p* < 0.01), TNF‐α (all *p* < 0.001), IL‐2 (all *p* < 0.01), and IL‐4 (*p* < 0.001 vs. PBS, *p* < 0.01 vs. LAIV and IIV) (Figure 5M–P). Representative flow cytometry images of antigen‐specific CD8^+^ IFN‐γ^+^, CD8^+^ TNF‐α^+^, CD8^+^ IL‐2^+^, CD8^+^ IL‐4^+^, CD4^+^ IFN‐γ^+^, CD4^+^ TNF‐α^+^, and CD4^+^ IL‐2^+^ CD4^+^ IL‐4^+^ T cells are shown in Figure S3. These cytokine‐producing cells were identified within the CD3^+^ CD4^+^ or CD3^+^ CD8^+^ T‐cell populations using flow cytometry gating strategies, as detailed in Figure S4. Collectively, these findings demonstrate that the mosaic H1N1 LAIV elicits robust homologous and heterologous antigen‐specific CD4^+^ and CD8^+^ T‐cell responses after immunization.

### 2.7 Intranasal Mosaic H1N1 LAIV Elicits T_RM_ Cells and B_RM_ Cells in the Respiratory Tract

**FIGURE 6:**
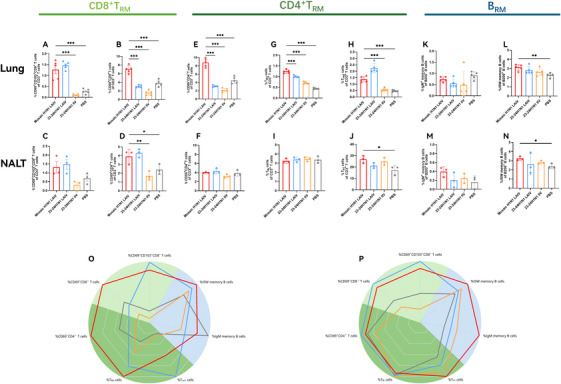
The mosaic H1N1 LAIV elicits CD8^+^ and CD4^+^ T_RM_ and B_RM_ cells in the lungs and NALT. The lungs and NALT were collected on Day 35 postimmunization and evaluated by flow cytometry. (A–D) Percentages of CD69^+^ CD8^+^ T cells and CD69^+^ CD103^+^ CD8^+^ T cells, which represent CD8^+^ T_RM_ cells, in the lungs (A and B) and NALT (C and D). (E–J) Percentages of CD69^+^ CD4^+^ T cells and subsets of T_RH_ cells (PD‐1^hi^ PSGL1^lo^ FR4^hi^ CD4^+^ T cells) and T_rm1_ cells (PD‐1^hi^ PSGL1^hi^ FR4^lo^ CD4^+^ T cells), which represent CD4^+^ T_RM_ cells in the lungs (E, G, and H). Percentages of CD69^+^ CD4^+^ T, T_fh_ (PSGL1^lo^ FR4^hi^ CD4^+^ T cells) and T_h1_ (PSGL1^hi^ FR4^lo^ CD4^+^ T cells) cells, representing CD4^+^ T_RM_ cells, in the NALT (F, I, and J). (K–N) Percentages of IgM^+^ memory B cells (IgM^+^ IgD^−^ CD38^+^ CD19^+^ CD138^−^) and ISW memory B cells (IgM^−^ IgD^−^ CD38^+^ CD19^+^ CD138^−^), which represent B_RM_ cells, in the lungs (K and L) and NALT (M and N). (O and P) Radar plots summarizing the comprehensive expression profiles of CD8^+^ and CD4^+^ T_RM_ and B_RM_ cells in the lungs (O) and NALT (P). The production levels of CD8 T_RM_ cells, CD4 T_RM_ cells, and B_RM_ cells are represented by light green, dark green, and blue, respectively. The data are expressed as the means ± SDs. Statistical analysis was performed via a nonparametric Kruskal–Wallis test or one‐way ANOVA, with significance indicated as *p < 0.05, **p < 0.01, and ***p < 0.001 (*n* = 5 mice per group for the lungs and *n* = 3 sample/9 mice per group for the NALT). The data are presented as bar charts with individual data points.

The generation of tissue‐resident memory B (B_RM_) and T (T_RM_) cells is crucial for rapid local recall responses and long‐term protection against respiratory pathogen infection such as influenza viruses [34, 35]. We analyzed the CD4^+^, CD8^+^ T_RM_ and B_RM_ populations in the NALT and lungs on Day 35. Intravascular staining with an anti‐CD45 mAb was performed 5 min prior to euthanasia to exclude circulating leukocytes. In the lungs, the mosaic H1N1 LAIV significantly increased the percentages of CD69^+^ CD8^+^ T cells (all *p* < 0.001 vs. IIV and PBS) (Figure 6A) and CD69^+^ CD103^+^ CD8^+^ T cells (all *p* < 0.001) (Figure 6B). In the NALT, we observed a slight, but not significant increase in the percentage of CD69^+^ CD8^+^ T cells (Figure 6C) and a significant increase in the percentage of CD69^+^ CD103^+^ CD8^+^ T cells (*p* < 0.01 vs. IIV, *p* < 0.05 vs. PBS) (Figure 6D).

CD4^+^ T_RM_ cells are typically CD69^+^ CD103^−^ [36]. Vaccination with the mosaic H1N1 LAIV drove significant lung‐specific accumulation of CD69^+^ CD4^+^ T cells (all *p* < 0.001) (Figure 6E), whereas no significant differences were observed in NALT across the groups (Figure 6F). Recent studies have classified lung CD4^+^ memory T cells as T_RH_ (T_fh_‐like) and T_rm1_ (T_h1_‐like) cells, characterized by the phenotype PD‐1^hi^PSGL1^lo/hi^FR4^hi/lo^ [37, 38, 39]. T_RH_ cells, a subset of CD4^+^ T_RM_ cells, play important roles in promoting CD8^+^ T‐ and B‐cell responses in nonlymphoid organs, further enhancing local immune protection [40]. In the lungs, the mosaic H1N1 LAIV induced significantly greater percentages of T_RH_ cells (all *p* < 0.001) and T_rm1_ cells (*p* < 0.001 vs. IIV and PBS) (Figure 6G,H). In the NALT, the percentage of T_h1_ cells was higher (*p* < 0.05 vs. PBS), while T_fh_ cells did not differ among all the groups (Figure 6I,J).

B_RM_ cells are important components of the immune response to respiratory viruses [35]. Intranasal administration elicited robust expression of lung and nasal‐resident B_RM_ cells [41, 42]. Here, we gated IgM^+^ memory B cells and isotype‐switched (ISW) memory B cells. The mosaic H1N1 LAIV significantly increased the percentages of ISW memory B cells in the lungs (*p* < 0.01 vs. PBS) and NALT (*p* < 0.05 vs. PBS), while no significant increase in IgM^+^ memory B cells was observed in the lungs or NALT (Figure 6K–N). Radar plots were generated to comprehensively and intuitively visualize the secretory profiles of tissue‐resident T and B cells in the lungs and NALT (Figure 6O,P). Representative flow cytometry results for CD69^+^ CD8^+^ T, CD69^+^ CD103^+^ CD8^+^ T, CD69^+^ CD4^+^ T, T_RH_, T_rm1_, IgM^+^ memory B, and ISW memory B cells in the lungs are shown in Figure S5. Similarly, representative flow cytometry results for CD69^+^ CD8^+^ T, CD69^+^ CD103^+^ CD8^+^ T, CD69^+^ CD4^+^ T, T_fh_, T_h1_, IgM^+^ memory B, and ISW memory B cells in NALT are also shown in Figure S5. The gating strategies for both the lungs and NALT are presented in Figure S6. These data highlight that mosaic H1N1 LAIV vaccination increased the abundance of CD8^+^ and CD4^+^ T_RM_ cells and that of B_RM_ cells in the lungs and NALT.

### 2.8 Intranasal Mosaic H1N1 LAIV Fully Protected Mice Against H1N1 Virus Challenge

**FIGURE 7:**
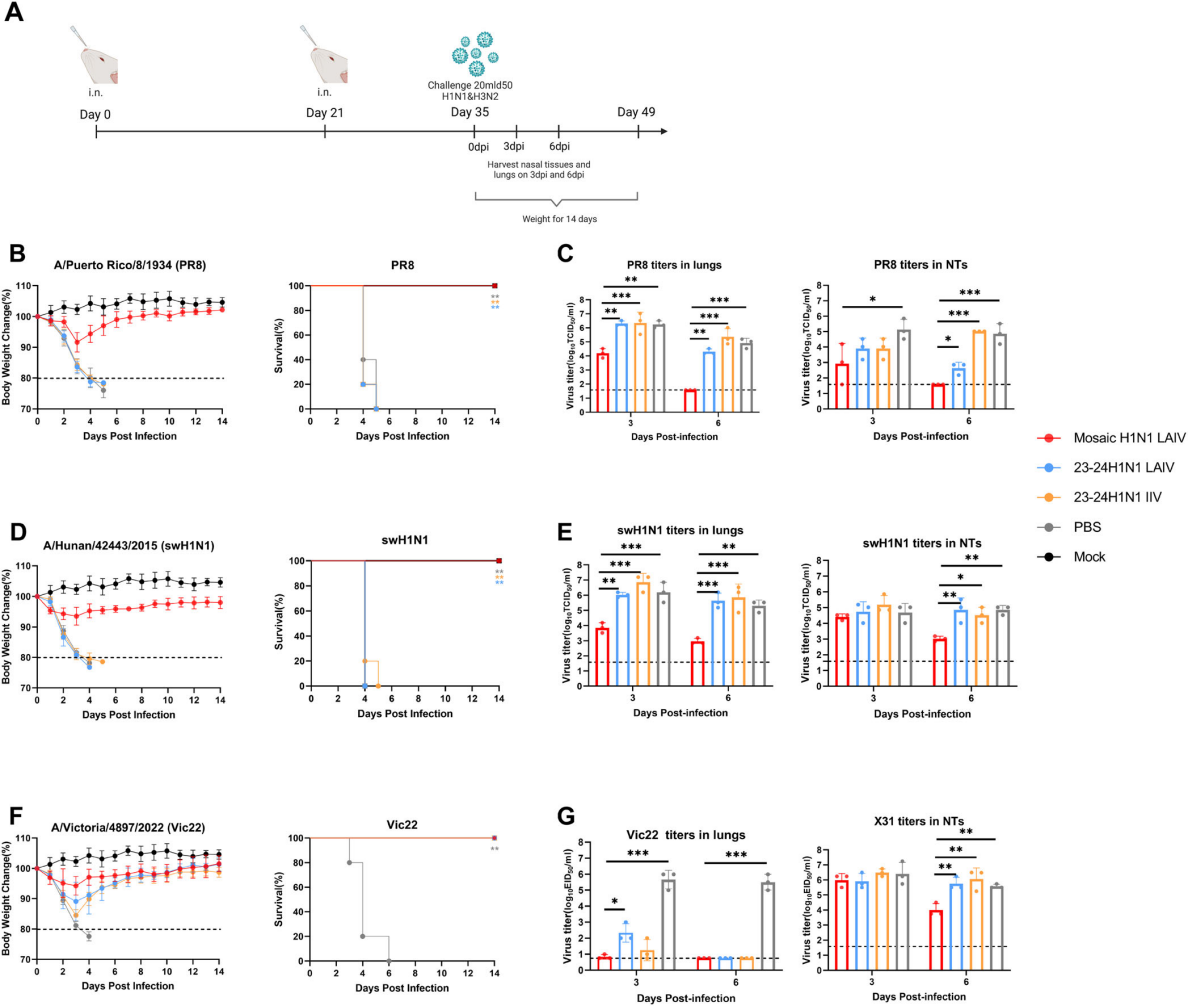
Intranasal vaccination protected mice against homologous virus challenge. (A) Experimental design for the challenge studies. The mice were immunized at Week 0 and Week 3 and then challenged with three distinct H1N1 viruses at 20 MLD_50_ at Week 5. (B–G) Body weight changes (*n* = 5 mice per group), survival rates (*n* = 5 mice per group) and viral titers in the lungs and NTs (*n* = 3 mice per group) were assessed on Day 35 in mice challenged with A/Puerto Rico/8/34 (PR8) (B and C), A/Hunan/42443/2015 (swH1N1) (D and E), and A/Victoria/4897/2022 (Vic22) (F and G). Body weight changes (dotted lines below 80% indicate the humane endpoint) and survival were monitored for 14 days. The data are expressed as the means ± SDs. Statistical analysis was performed via the log‐rank test (B, D, and F) and one‐way ANOVA (C, E, and G), with significance indicated as *p < 0.05, **p < 0.01, and ***p < 0.001. Panels (B), (D), and (F) are presented as line charts. Panels (C), (E), and (G) are presented as bar charts with individual data points.

To evaluate the broad‐spectrum protective response after mosaic H1N1 LAIV vaccination, we first conducted a cross‐protective response within the subtype. Three H1N1 strains with low‐homology were selected, including the ancestral strain A/Puerto Rico/8/34 (PR8), the swine virus A/Hunan/42443/2015 (swH1N1), and the seasonal influenza virus A/Victoria/4897/2022 (Vic22) (the WHO‐recommended 23–25 seasonal H1N1 viruses were not included in our Mosaic Algorithm Library). Mice were challenged with a high dose of 20 MLD_50_ (50% mouse lethal dose) to verify the potent protective effect of the mosaic H1N1 LAIV. Challenges were performed 14 days after double‐dose intranasal vaccination in all groups. Protection was assessed based on body weight loss and survival rates over 14 days, viral titers in the lungs and NTs, and lung histopathology at 3 and 6 days postinfection (dpi) (Figure 7A).

In the PR8 challenge group, mosaic H1N1 LAIV‐immunized mice exhibited 100% survival (all *p* < 0.01) with mild weight loss (<10%) and fully recovered within 14 days, while mice in the control groups experienced 100% mortality within 5 days (Figure 7B). Viral loads in the lungs and NTs were analyzed at 3 and 6 dpi. The mosaic H1N1 LAIV group presented significantly less viral shedding in the lungs at both 3 and 6 dpi (*p* < 0.01 vs. LAIV, *p* < 0.001 vs. IIV and PBS). Viral shedding in the NTs of the mosaic H1N1 LAIV group was lower at 3 dpi (*p* < 0.05 vs. PBS) and significantly lower at 6 dpi (*p* < 0.05 vs. LAIV, *p* < 0.001 vs. IIV and PBS) (Figure 7C). Histological analysis revealed no obvious lesions in the lungs of the mice in the mosaic H1N1 LAIV group, with uniformly arranged alveolae and intact alveolar septa. In contrast, mice in the three control groups presented bronchial epithelial damage, inflammatory cell infiltration in the interstitium, and evident congestion (Figure S7).

In the swine influenza virus challenge group, the mosaic H1N1 LAIV‐immunized mice exhibited 100% survival (all *p* < 0.01) with mild weight loss (<10%), whereas mice in all the other groups experienced 100% mortality within 5 days (Figure 7D). Viral shedding levels of lungs and NTs were similar to those observed in the PR8 challenge group (Figure 7E). Additionally, the mosaic H1N1 LAIV‐immunized mice exhibited minimal cellular infiltration and inflammation (Figure S8).

A/Victoria/4897/2022 was also applied to challenge mice vaccinated with the 23–24H1N1 LAIV and 23–24H1N1 IIV. Mosaic H1N1 LAIV‐immunized mice achieved 100% survival (*p* < 0.01 vs. PBS) with mild weight loss (<10%) and fully recovered within 14 days (Figure 7F). Notably, the mosaic H1N1 LAIV group experienced less weight loss than the 23–24H1N1 LAIV/IIV groups did, whereas the negative control group experienced 100% mortality within 4 days. In the lungs and NTs, the mosaic H1N1 LAIV group presented significantly reduced viral shedding at both 3 and 6 dpi (*p* < 0.001 vs. PBS in lungs, *p* < 0.01 vs. PBS in NTs) (Figure 7G). All the groups except the PBS group presented little or no pathological damage (Figure S9). These results demonstrate that the mosaic H1N1 LAIV provides complete protection against lethal homologous influenza virus challenge, including challenge with strains not included in the Mosaic Algorithm Library.

### 2.9 Intranasal Mosaic H1N1 LAIV Fully Protected Mice Against H3N2 Virus Challenge

**FIGURE 8:**
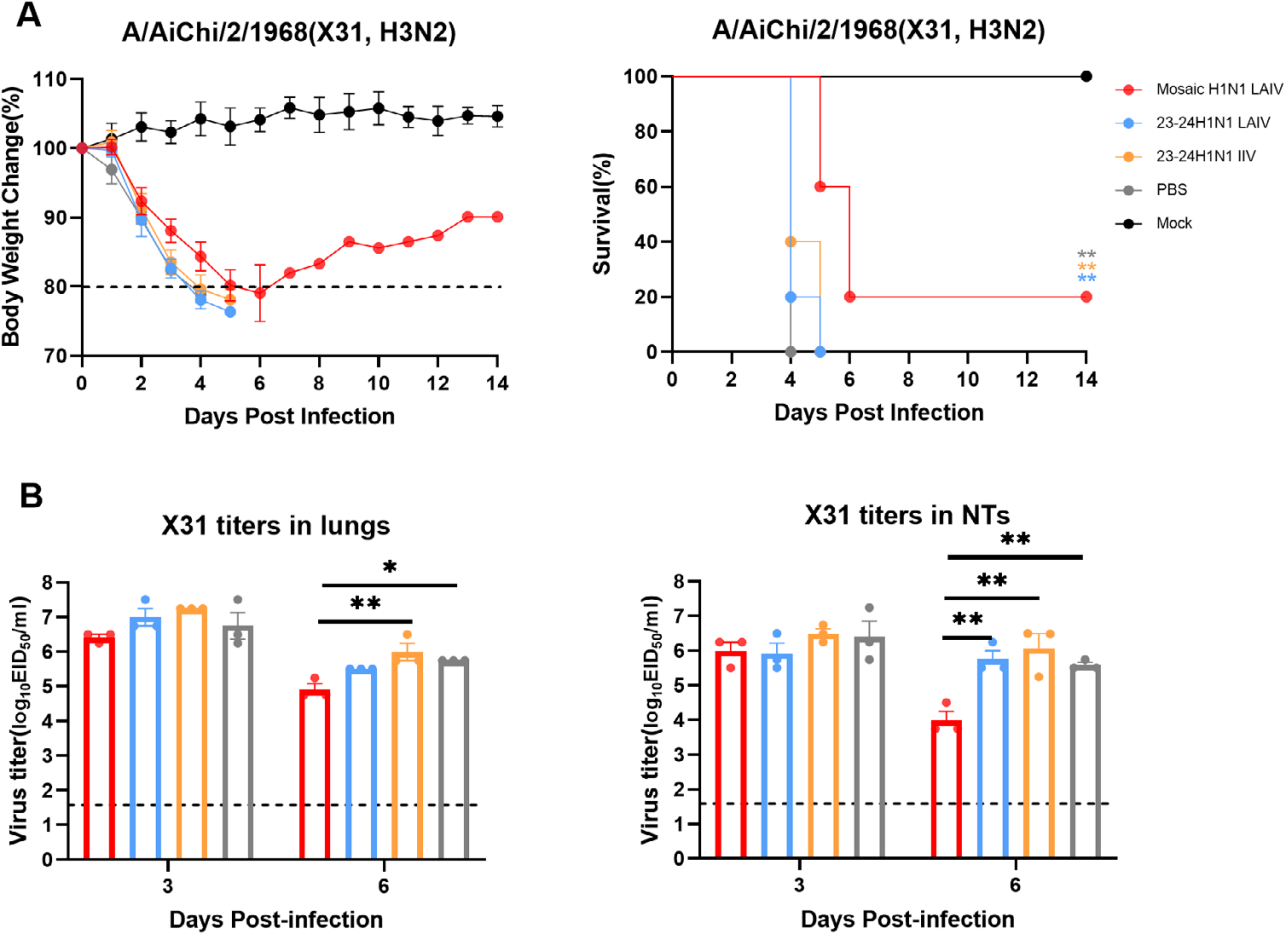
Intranasal vaccination protected mice against heterologous virus challenge. Body weight changes and survival rates (*n* = 5 mice per group) (A) and viral titers in the lungs and NTs (*n* = 3 mice per group) (B) were assessed in mice challenged with 20 MLD50 group 2 strain A/Aichi/2/1968 (X31, H3N2) on Day 35. The data in panel (A) are presented as a line chart. The data in panel (B) are presented as a bar chart with individual data points. The data are presented as the means ± SDs. Statistical significance was determined via the log‐rank test (A) and one‐way ANOVA (B), with significance levels indicated as *p < 0.05, **p < 0.01, and ***p < 0.001. Body weight changes (dotted lines below 80% indicate the humane endpoint) and survival were monitored for 14 days.

To evaluate the broad‐spectrum protection of the mosaic H1N1 LAIV across subtypes, all groups were challenged with 20 MLD_50_ of the group 2 heterologous ancestral strain A/Aichi/2/1968 (X31, H3N2). The mosaic H1N1 LAIV group showed a survival rate of 20% (all *p* < 0.01), whereas the mortality rate in the control groups reached 100% within 5 days (Figure 8A). The mosaic H1N1 LAIV group presented significantly reduced viral shedding in the lungs at both 3 dpi (*p* < 0.01 vs. IIV, *p* < 0.05 vs. PBS) and 6 dpi (*p* < 0.01 vs. LAIV, IIV, and PBS) (Figure 8B), along with milder cellular infiltration and inflammation (Figure S10). In contrast, the 23–24H1N1 LAIV/IIV mice presented viral shedding and tissue inflammation comparable to those in the PBS group. No significant differences in viral load were observed in the NTs among all groups. These results demonstrate that the mosaic H1N1 LAIV provides some degree of protection against lethal heterologous group 2 influenza virus challenge, highlighting its potential as a broad‐spectrum influenza LAIV.

## 3 Discussion

The LAIV, as the only clinically approved mucosal influenza vaccine, has been proven to be safe and provide consistent immunogenicity through large‐scale validation. However, the current seasonal influenza vaccine has limitations because of the high variability of influenza viruses. The long production process based on WHO‐recommended strains may not match the actual circulating strains, reducing vaccine efficacy and increasing pandemic risk. This study aimed to design a broad‐spectrum live attenuated vaccine that can elicit cross‐reactive immune responses and offer cross‐protection against different influenza strains.

Mosaic design, with certain advantages such as broader antigenic coverage and extensive immune responses, has been applied in the design of highly variable viruses such as HIV, dengue virus, and influenza [19, 21, 22, 23, 43]. By using attenuated live vaccine technology and the mosaic antigen design platform, we developed a mosaic H1N1 LAIV. This recombinant virus integrates the mosaic nucleotide sequences mH1 and mN1 into a ca, attenuated master donor strain. In vitro studies confirmed its stability, high titers and attenuation, whereas in vivo trials demonstrated its safety, immunogenicity, and protective efficacy.

Bioinformatics analysis revealed that 09–22H1m and 09–22N1m contain more conserved and broadly distributed T‐cell epitopes than the latest WHO‐recommended strains do. Antigenic comparisons with hundreds of influenza A virus strains revealed that these strains share most of their epitopes, which supports the experimental results concerning broad cross‐reactivity. Notably, although the epitope coverage of X31 (H3N2) was relatively low, the vaccine still provided a certain level of protection against this strain. This could be because the specific covered sites have significant immunological implications; thus, future research should focus on these sites. Alternatively, it is possible that the actual effective coverage sites are not included in the current epitope library.

We conducted an evaluation of antibody production in vivo. The vaccine exhibited exceptional serological performance. Although studies have shown that the HAI response to the LAIV is weaker than that to intramuscular vaccines such as IIVs [44], our experimental results revealed that the mosaic H1N1 LAIV generated more robust and broader HAI and MN antibody responses than the commercial inactivated monovalent vaccine. In particular, the vaccine elicited strong responses against the cross‐host swine influenza strain A/Hunan/42443/2015 (swH1N1), an EA lineage closely related to the 2009 pandemic strain and recent epidemic strains, making it a valuable model for pandemic preparedness. While the mosaic H1N1 LAIV did not show statistically significant differences in HAI and MN levels for Vic19 or Vic22 compared with the other groups, this may be attributed to the high degree of dispersion and the use of one‐way ANOVA for statistical analysis. Nevertheless, the vaccine still generated HAI antibody levels greater than 40 and detectable neutralizing antibody titers against these strains. Notably, Vic22 is the latest WHO‐recommended H1N1 vaccine strain for the Northern Hemisphere for 2023–2025. It was not included in the mosaic library, which was constructed using sequences up to October 31, 2022. Vic22 was first submitted to Sharing All Influenza Data (GISAID) (ID: EPI_ISL_16003490) on December 5, 2022, after the library cutoff date. Additionally, the mosaic H1N1 LAIV also generated partial HAI levels against heterologous strains such as H9N2 and H5N1, indicating its broad serological coverage against group 1 strains. However, it did not elicit effective serological levels against the PR8 strain or heterologous H3N2, likely because of significant differences in the conformational epitopes of the HA protein.

Notably, the mosaic H1N1 LAIV demonstrates remarkable broad‐spectrum mucosal immunity. The vaccine elicited significant IgA responses in both the lungs and NALF, targeting not only recent H1N1 strains from the past 15 years but also the historic PR8 strain and the swine influenza virus. Additionally, the mosaic H1N1 generated moderate levels of IgA antibodies against representative H5N1, H9N2, and Group 2 (H3 subtype) viruses, especially the ancestral strain X31. By inducing strong IgA responses in both the upper and lower respiratory tract, this vaccine provides dual protection against initial viral invasion in the nasal mucosa and severe lung infections in the lower respiratory tract. These results further indicate that the mosaic H1N1 LAIV provides cross‐protection against both group 1 and 2 influenza viruses, offering potential defenses against future seasonal influenza and epidemic strains.

We demonstrated that the mosaic H1N1 LAIV significantly enhanced Th1, Th2, and Th17 immune responses against the low‐homology PR8 strain and the heterologous historic H3N2 strain X31. Specifically, the vaccine elicited PR8‐specific CD4⁺ and CD8⁺ T‐cell responses characterized by the production of Th1‐associated cytokines, including IFN‐γ, IL‐2, and TNF‐α. In contrast, X31‐specific CD4⁺ and CD8⁺ T‐cell responses included both Th1‐associated cytokines (IFN‐γ, IL‐2, and TNF‐α) and the Th2‐associated cytokine IL‐4. This broad immune response may be attributed to the mosaic H1N1 LAIV covering a broader range of T‐cell linear epitopes. CD8⁺ T cells directly eliminate influenza‐infected cells through cytotoxicity, while CD4⁺ T cells contribute to viral clearance, differentiate into Th1/Th2/Th17 subsets, support B‐cell antibody production, and activate CD8⁺ T cells to establish long‐term memory [33, 45, 46, 47]. This comprehensive immune activation enables the induction of robust cellular immune responses against mismatched strains [48].

Intranasal vaccination is particularly effective at recruiting resident memory cells to the upper and lower respiratory tracts, thereby boosting local immunity. T_RM_ cells permanently reside on mucosal surfaces such as the lungs and nasal cavities, providing a first line of defense against reinfection and accelerating pathogen clearance. Numerous studies have confirmed the importance of CD4^+^ and CD8^+^ T_RM_ cells in local antiviral immunity [49, 50, 51, 52, 53, 54]. Similarly, B_RM_ cells respond rapidly to respiratory viral infections and provide long‐lasting protection [35, 55, 56]. In this study, we analyzed the presence of T_RM_ and B_RM_ cells in the lungs and NALT of mice. Our results demonstrated that the mosaic H1N1 LAIV significantly increased CD8^+^ T_RM_ cell abundance in both the lungs and NALT. In the lungs, the vaccine also markedly increased the number of CD4^+^ T_RM_ cells, particularly the T_RH_ and T_rm1_ subsets. With respect to B_RM_ cells, in both the lungs and NALT, we observed a significant increase in the number of ISW memory cells, while the number of IgM⁺ memory B cells was relatively low, similar to that in the PBS group. This may have occurred because, at the time of sampling on Day 35 after primary immunization, the immune response had already entered a mature stage, indicating that memory B cells had undergone class switching during the immune response, which transitioned from an early IgM‐dominant phase to a phase dominated by IgA or IgG [57, 58]. In the PBS control group, although no specific antigen stimulation was administered, nonspecific immune activation or environmental exposure (such as to microbes or endogenous antigens) might have induced a certain level of IgM⁺ memory B cells. Since we measured the overall memory B‐cell levels, this nonspecific activation could have led to an elevated baseline level of IgM⁺ memory B cells. When we compared the numbers of memory T and B cells between the NALT and the lungs, we found that although the overall numbers of these memory cells in the NALT were significantly or slightly greater than those in the control and IIV groups, the numbers were still smaller than those in the lungs. The same phenomenon was observed for IgA detection. In fact, the presence of T_RM_ and B_RM_ cells in the NALT is highly important. Like human tonsils (the equivalent of NALT in humans), approximately 65% of T cells within the NALT of mice exhibit a memory phenotype [59]. Research has demonstrated that T_RM_ cells in the upper respiratory tract not only provide long‐term protection but also effectively limit the transmission of the influenza virus from the upper to the lower airways [54]. The relatively narrow upper respiratory tract in mice may lead to a shorter antigen retention time in the nasal mucosa, which could be related to the observed immune cell distribution. Notably, although the immune responses in NALT were relatively modest, the dominant induction of T_RM_/B_RM_ cells in the lungs implies the prioritization of protection against lower respiratory complications. In the future, we plan to validate these findings using ferret or human nasal mucosa samples.

The results of protection experiments revealed that the mosaic H1N1 LAIV provides complete protection against high‐dose lethal challenges from an ancient highly virulent strain, zoonotic strains, and the Vic22 strain, which was not included in the design library. Additionally, the vaccine conferred partial protection against the heterologous group 2 strain. Increased viral clearance rates were observed in the lungs and NTs, along with no obvious or only mild changes in lung tissue. These findings indicate that the mosaic H1N1 LAIV offers both local and systemic protection. Specifically, it effectively controls infection at an early stage, significantly inhibits viral replication and transmission, mitigates mild symptoms, and reduces the risk of severe illness. Our experiments confirmed that protective immunity can be generated even in the absence of strain‐specific serum antibodies, which is likely mediated by antigen‐specific IgA, antigen‐specific T cells, and T_RM_ and B_RM_ cells, indicating that both systemic and local mucosal immune responses are important for protection [51, 60, 61, 62].

However, our study has certain limitations. In addition to the limitation posed by the narrow upper respiratory tract of mice, which may not fully recapitulate human infection dynamics, we did not evaluate the durability of the vaccine‐induced immune response, as such experiments were constrained by the relatively short lifespan of the mice. To address these limitations, we plan to conduct future studies using ferret models, as ferrets have a longer lifespan and more closely mimic human respiratory tract anatomy and immune responses. Furthermore, we are actively exploring strategies to dynamically update the mosaic library on the basis of real‐time surveillance data, ensuring better alignment with circulating strains. Additionally, future work will include investigations into the mechanisms of the mosaic vaccine, such as T‐cell exhaustion and lung lavage passive transfer experiments, as well as experimental validation of the mosaic antigens against newly identified influenza viruses to further assess their efficacy and protective capability. These efforts aim to increase the broad‐spectrum potential of the mosaic H1N1 LAIV and its ability to overcome emerging influenza threats.

In summary, the mosaic H1N1 LAIV, with its broad coverage of T‐cell epitopes, was designed to induce broad cross‐reactive immunity and protection against homologous and heterologous influenza A virus strains, addressing the limitations of traditional vaccines that require annual strain updates. The results of this study revealed that the mosaic H1N1 LAIV elicited multiple robust immune responses, including the following: (i) strong cross‐reactive HI and MN antibody responses; (ii) high‐level, broad cross‐reactive IgA antibodies; (iii) antigen‐specific systemic CD4^+^ and CD8^+^ T‐cell responses; (iv) CD8^+^ and CD4^+^ T_RM_ and B_RM_ cells in the upper and lower respiratory tracts; and (v) robust cross‐protection against both homologous and heterologous strains. Like traditional vaccines, the mosaic H1N1 LAIV has significant potential for large‐scale production. As mentioned earlier, it can achieve a titer of 8.9 log_10_ EID_50_/mL in the P4 generation, which is comparable to that of clinically approved vaccines [63, 64]. Its attenuated backbone has been clinically validated, and it holds great clinical potential compared with other broad‐spectrum influenza vaccines. However, unlike traditional vaccines, it eliminates the need for annual strain updates, thereby substantially increasing vaccine effectiveness. When lyophilized, it has excellent stability, maintaining potency for up to 9 months [65]. Furthermore, the strategy used to develop the mosaic H1N1 LAIV is currently being applied to create mosaic H3N2 and B/Victoria (BV) LAIV candidates. In the future, a trivalent broad‐spectrum live attenuated vaccine comprising mosaic H1N1, H3N2, and BV LAIVs could provide optimal protection against a wide range of influenza strains, offering a comprehensive and clinically valuable solution for influenza prevention.

## 4 Materials and Methods

### 4.1 Design and Evaluation of the Mosaic Sequence

The HA and NA amino acid sequences of H1N1 human influenza A viruses isolated between January 1, 2009 and October 31, 2022 were downloaded from the GISAID EpiFlu database and the National Center for Biotechnology Information (NCBI) databases. The design methodology followed previously established approaches [20]. In brief, redundant and nonnatural sequences were removed using the tools BioAider and BioEdit. Multiple sequence alignment of the original viral sequences was performed using MAFFT. Finally, a genetic algorithm was used to design mosaic HA and NA proteins of H1N1, with the goal of maximizing T‐cell epitope coverage. The T‐cell epitopes included in the mosaic design were based on human leukocyte antigen molecules, ensuring considerable relevance to human immune responses [18]. Finally, AlphaFold3 (DeepMind, https://alphafoldserver.com) was used to predict the structure of the mosaic HA and NA proteins, which are referred to as 09–22H1m and 09–22N1m, respectively.

The Epitope Coverage Assessment Tool (Epicover, https://www.hiv.lanl.gov/content/sequence/MOSAIC/epicover.html) and the Positional Epitope Coverage Assessment Tool (Posicover, https://www.hiv.lanl.gov/content/sequence/MOSAIC/posicover.html) were utilized for epitope coverage analysis. The background protein dataset used for testing was larger than that used for mosaic design, incorporating sequences from January 1, 2009 to December 31, 2024. This dataset comprises a total of 22,375 full‐length H1N1 HA and 16,774 full‐length H1N1 NA amino acid sequences from natural influenza virus strains, with duplicate sequences removed. The HA and NA proteins of A/Victoria/4897/2022, along with the 09–22 H1m and 09–22N1m proteins, were selected as target antigens for comparative predictions. All analyses were performed with the default settings.

For epitope comparison analysis, Visual Studio Code (VSCode) was used. The control dataset included 168 representative influenza A virus strains, including all known H1N1 lineages available in GISAID (*n* = 139), ancestral nonlineage H1N1 strains (*n* = 15), animal‐derived H1N1 strains (*n* = 9), and other influenza A virus strains used in the experiments (*n* = 5). Representative H1N1 strains were selected from available isolates (1934–present) by removing highly repetitive sequences and ensuring maximal sequence diversity (geographic, temporal, and host species coverage). The HA and NA sequences of these strains were compared with those of 09–22H1m and 09–22N1m, respectively.

### 4.2 Cells and Influenza Viruses

Human embryonic kidney 293T cells (HEK293T cells, RRID: CVCL_0063) and Madin–Darby canine kidney cells (MDCK cells, RRID: CVCL_0422) were cultured in Dulbecco's modified Eagle's medium (Gibco) supplemented with 10% fetal bovine serum (FBS; Gibco) and 1% penicillin–streptomycin (PS; Gibco) at 37°C with 5% CO_2_.

All provided influenza A viruses were propagated in 9‐day‐old SPF embryonated eggs at 37°C for 48 h, harvested, aliquoted, and stored at –80°C. The viral titers were determined and expressed as EID_50_. All experiments concerning influenza viruses were performed in biosafety level 2 (BSL2) facilities. Complete information on the strains and the corresponding recommended years by the WHO is provided in Supplementary Data 2.

To generate the PR8 and X31 purified viruses, β‐propiolactone was added to the viral allantoic fluid to achieve a final concentration of 0.05%, followed by overnight incubation at 4°C. Complete inactivation was confirmed by chicken embryo passage. The viral particles were then separated and enriched using sucrose density gradient ultracentrifugation and subsequently dissolved in PBS for storage and further use.

### 4.3 Generation of the Mosaic LAIV

To generate the mosaic virus, we optimized the 09–22H1m/N1m amino acid sequences and determined the corresponding nucleotide sequences. The noncoding regions were selected from the most conserved and commonly used sequences of H1N1 in nature to ensure stability and functionality, and the coding regions were obtained through BLAST alignment of the amino acid sequences to ensure biological activity and natural adaptability. Specifically, mH1 was derived from the HA sequence of A/Thailand/CU‐MV8/2010 (GenBank: ADK26546.1), and mN1 was derived from the NA sequence of A/Minnesota/08/2013 (GenBank: AIM39399.1). These sequences, along with the HA and NA sequences of A/Victoria/4897/2022 and the other six gene segments of Len/17, were synthesized by Beijing Junyi Yinghao Biotechnology Co., Ltd., and subsequently cloned and inserted into the pHW2000 plasmid.

The mosaic H1N1 LAIV and 23–24H1N1 LAIV were rescued via reverse genetics technology. In brief, HEK293T (3 × 10⁵) and MDCK (1 × 10⁵) cells were plated into six‐well plates and grown until they reached 70–90% confluence. The transfection system for one well is as follows: 500 ng of each plasmid was combined with 200 µL of Optimized Minimal Essential Medium (Opti‐MEM; Gibco) and 10 µL of FUGENE HD (Promega). After a 10‐min incubation, 800  µL of Opti‐MEM was added to the mixture. The medium in the well was removed and replaced with the prepared transfection mixture. The cells were then incubated at 33°C in a 5% CO_2_ incubator for 24 h. Next, virus maintenance medium was added to each well (1 mL of Opti‐MEM containing 4 µg/mL TPCK‐trypsin (Sigma) and 1% PS (100 U/mL, Gibco) for one well), and the cells were further incubated at 33°C and 5% CO_2_ for an additional 48 h.

At 72 h after transfection, the culture medium was harvested and inoculated into 9‐day‐old SPF embryonated eggs for viral amplification. The chicken embryos were incubated at 33°C for 48 h [66]. Once hemagglutination titers were measurable, the allantoic fluids from the chicken embryos were collected, and the virus sequences were confirmed by Sanger sequencing.

### 4.4 TCID50 and EID50 Assays

To determine the TCID_50_, MDCK cells were first trypsinized to obtain a uniform single‐cell suspension and seeded into 96‐well plates at a density of 3 × 10⁴ cells per well. After incubation for 24 h at 37°C in a humidified incubator with 5% CO_2_ to allow cell attachment, the viral allantoic fluid was diluted at a ratio of 1:100 (tissue samples were diluted starting from the undiluted stock), followed by a series of half‐log serial dilutions. Subsequently, 30 µL of the diluted virus samples were added to the preseeded MDCK cells. After 1 h of incubation, each well received an addition of 170 µL of the virus maintenance medium, with each sample being tested in quadruplicate. After 48 h of incubation at 37°C, the culture supernatants were collected from each well and subjected to a hemagglutination assay using 1% (v/v) turkey red blood cells. Wells positive for hemagglutination were recorded, and the TCID_50_ value was calculated using the Reed–Muench method [67].

To determine the EID_50_, viral allantoic fluid was serially diluted 10‐fold in PBS. Each dilution (100 µL) was inoculated into the allantoic cavity of 9‐day‐old SPF embryonated chicken eggs (five eggs per dilution). The eggs were incubated at 37°C (33°C for the mosaic LAIV) and candled daily to monitor embryonic viability. After 48 h, the allantoic fluid was harvested from each egg, and the presence of the virus was tested by a hemagglutination assay using 1% turkey red blood cells. The EID_50_ value was calculated according to the Reed–Muench method.

### 4.5 Biological Characteristics

The ts and ca phenotypes were assessed on the basis of the EID_50_ titers of the rescued candidate viruses measured at 29, 33, and 39°C. A virus was classified as having a ts phenotype if its titer at 39°C was at least three log_10_ EID_50_ lower than that at 33°C. Similarly, a virus was considered cold adapted if its titer at 29°C was no more than three log_10_ EID_50_ lower than that at 33°C [24, 25].

Images of mosaic H1N1 LAIV particles were obtained via TEM. The samples were allowed to absorb onto Formvar and carbon‐coated grids for at least 1 min, stained with 1% (w/v) phosphotungstic acid (pH 6.8) for 30 s, and air‐dried. The grids were then observed under a Tecnai G2 Spirit BioTWIN transmission electron microscope (FEI, Eindhoven, Netherlands), and images were acquired using Gatan Digital Micrograph software.

### 4.6 In Vivo Studies

Sample sizes were selected empirically based on observed variability and the statistical power required, with consideration given to minimizing animal use.

To evaluate the safety profile of the mosaic virus in mice, 6–8‐week‐old SPF female BALB/c mice (bred by the Animal Experiment Center of the National Institutes for Food and Drug Control, China) were randomly divided into four groups. The mice were intranasally (i.n.) administered P4‐generation mosaic H1N1 LAIV at a dosage of 10^7^ EID_50_ in a 30 µL volume. This dose was chosen on the basis of prior studies of live attenuated influenza A vaccines [64, 68, 69, 70]. Our preliminary experiments revealed stronger HAI antibody responses at the 10^7^ EID_50_ dose than at 10^5^–10^6^ EID_50_ dose (results not included). The control groups were categorized into three subsets: animals in one subset were immunized i.n. with commercial monovalent LAIV (23–24H1N1 LAIV) at the same dose; those in another subgroup were injected intramuscularly (i.m.) with the commercial monovalent IIV (23–24H1N1 IIV) at a dose of 1.5 µg/50 µL; and those in the last subgroup received 30 µL of PBS (Gibco) i.n. On Days 3 and 6 postimmunization, tissues from the lung, turbinate, heart, liver, spleen, kidney, intestine, and brain were collected from each group (*n* = 3 mice per group). Viral titers were measured by the TCID_50_ method.

To evaluate the immunogenicity and protective efficacy of the mosaic H1N1 LAIV in mice, female SPF BALB/c mice (6–8 weeks) were randomly divided into four groups. A prime‐boost strategy with a 21‐day interval was used. Animals in the mosaic H1N1 LAIV group received 10^7^ EID_50_ in a volume of 30 µL i.n., whereas those in the 23–24H1N1 LAIV group received the same dose and volume i.n. Animals in the 23–24H1N1 IIV group were injected i.m. with 1.5 µg of HA. The mice in the PBS group received 30 µL of PBS i.n. The mice were weighed daily for 14 days after the initial immunization. On Day 35 (14 days postboost), NALF, BALF, serum, spleen, NALT, and lung samples were collected. NALF, BALF, serum, spleen, and lung samples were taken from each group (*n* = 5 mice per group). NALT samples were pooled from nine mice per group (three mice per sample) because of low lymphocyte yields. The remaining mice were challenged with 20 MLD_50_ representative group 1 strains H1N1 (A/Puerto Rico/8/1934 (PR8), A/Hunan/42443/2015 (swH1N1), A/Victoria/4897/2022 (Vic22)) or a representative group 2 strain H3N2 (A/Aichi/2/1968 (X31)) virus. Clinical signs, body weight changes, and mortality were monitored for 14 days, with euthanasia mandated for any mouse that exhibited ≥20% weight loss (*n* = 5 mice per group). Euthanasia was performed by cervical dislocation. Lungs and NTs were harvested on Days 3 and 6 postchallenge for measurement of viral titers using the TCID_50_/EID_50_ method (*n* = 3 mice per group).

### 4.7 HAI Assay

For HAI antibody measurement, serum samples from immunized mice were pretreated with a 1:3 dilution in receptor‐destroying enzyme (RDE; Denka Seiken, Japan) and incubated at 37°C for 18 h, after which the samples were heat‐inactivated at 56°C for 30 min. HAI assays were performed using 1% turkey erythrocytes for H1N1 and H9N2, guinea pig erythrocytes for H3N2, and horse erythrocytes for H5N1, with four hemagglutination units of virus following standard WHO protocols. The HAI titer was defined as the reciprocal of the highest serum dilution that completely inhibited hemagglutination. The HAI assay was used to evaluate the ancient virulent strain PR8, swine influenza virus A/Hunan/42443/2015 (swH1N1), and WHO‐recommended seasonal H1N1 vaccine strains (2009–2025), including A/Victoria/4897/2022 (Vic22), A/Victoria/2570/2019 (Vic19), A/Guangdong‐Maonan/SWL1536/2019 (Mao19), A/Brisbane/02/2018 (Bris18), A/Michigan/45/2015 (Mich15), A/California/7/2009 (CA07), and A/California/04/2009 (pdm09, CA04), as well as the heterologous group 1 strains A/Hongkong/1073/99 (H9N2, HK99) and A/Cambodia/R0405050/2007 (H5N1, Cam07) and group 2 strains A/Hongkong/2671/2019 (H3N2, HK19), A/Darwin/9/2021 (H3N2, Darwin21), and A/Aichi/2/1968 (X31, H3N2).

### 4.8 MN Assay

Neutralizing antibody titers were determined using serum samples pretreated as in the HAI assay, including incubation with RDE at 37°C for 18 h and subsequent heat inactivation at 56°C for 30 min. Serially diluted serum samples (starting from a 1:10 dilution) were mixed with 100 TCID_50_ of influenza virus at 37°C in 5% CO_2_ for 1 h. The virus–serum mixture was then added to MDCK cells (1.5 × 10^4^ cells/well) and incubated at 37°C in 5% CO_2_ for 18 h [28]. After the supernatant was removed, the cells were fixed with ice‐cold 80% acetone for 10 min and then washed three times with PBS. The cells were fixed and then successively treated with a monoclonal antibody specific for the influenza virus nucleoprotein (Sino Biological, China), followed by a horseradish peroxidase (HRP)‐conjugated goat anti‐rabbit IgG secondary antibody (Sino Biological), after which 3,3′,5,5′‐tetramethylbenzidine (TMB) substrate was added. The absorbance was measured at 450 nm using a Multiskan FC Microplate Photometer (Thermo Fisher). The MN assay was used to evaluate PR8, swH1N1, and WHO‐recommended seasonal H1N1 vaccine strains (2009–2025), including A/Victoria/4897/2022 (Vic22), A/Victoria/2570/2019 (Vic19), A/Guangdong‐Maonan/SWL1536/2019 (Mao19), A/Brisbane/02/2018 (Bris18), A/California/7/2009 (CA07), and A/California/04/2009 (pdm09, CA04).

### 4.9 Enzyme‐Linked Immunosorbent Assays

To quantify antigen‐specific IgA antibodies in BALF and NALF, enzyme‐linked immunosorbent assays (ELISAs) were performed. In brief, high‐binding polystyrene ELISA plates (Corning) were coated with 1 µg/mL standard antigen (NIBSC, UK) or an anti‐mouse IgA (α‐chain specific) antibody (Sigma–Aldrich, Germany) in ELISA coating buffer (Solarbio), and incubated overnight at 4°C. The plates were blocked with the blocking buffer (Solarbio) for 1 h at 37°C. The plates were washed five times and then incubated with diluted BALF, NALF, or the standard IgA calibrator for 1 h at 37°C.  Following further washing, the plates were incubated with a goat anti‐mouse IgA alpha chain (HRP) (Abcam) for 1 h at 37°C. Finally, the TMB substrate was added and allowed to react for 10–20 min. The absorbance was measured at 450 nm using a Scientific Multiskan microplate reader (Thermo). Sample concentrations were calculated by fitting the standard curve to a four‐parameter logistic model. ELISAs were performed with PR8, swH1N1, and the WHO‐recommended seasonal H1N1 vaccine strains (2009–2025), including A/Victoria/4897/2022 (Vic22), A/Victoria/2570/2019 (Vic19), A/Guangdong‐Maonan/SWL1536/2019 (Mao19), A/Brisbane/02/2018 (Bris18), A/Michigan/45/2015 (Mich15), and A/California/7/2009 (CA07), as well as the heterologous group 1 strains A/Hongkong/1073/99 (H9N2, HK99) and A/Cambodia/R0405050/2007 (H5N1, Cam07) and group 2 strains A/Hongkong/2671/2019 (H3N2, HK19), A/Darwin/9/2021 (H3N2, Darwin21), and A/Aichi/2/1968 (H3N2, X31).

### 4.10 T‐cell Surface Marker Analysis and Intracellular Cytokine Staining

The expression of cell markers and cytokines in T cells from single‐cell suspensions of lung, NALT or spleen tissue was assessed by flow cytometry. Five minutes before euthanasia, the mice were intravenously injected with 2 mg of CD45‐APC‐Cy7 (BD) in 200 µL of PBS via the tail vein to label circulating immune cells and allow their exclusion during flow cytometry analysis of tissue‐resident immune cells in the lungs and NALT.

The lungs and NALT were collected, minced, and digested with 1 mg/mL collagenase D (Roche) at 37°C for 1 h. Single‐cell suspensions were obtained by filtering the samples through a 70‐µm cell strainer and enriching them for lymphocytes with lymphocyte separation solution. The cells were stained with two different antibody panels. The first panel included antibodies against CD3ε‐BV605, CD4‐RB780, CD8α‐BV510, CD103‐BV786, CD45R/B220‐FITC, CD38‐BV421, CD138‐BV650, CD69‐APC, CD19‐PE, IgD‐BV711, and IgM‐PE‐Cy7 (BD Biosciences). The second panel included antibodies against CD3ε‐BV605, CD4‐RB780, CD162 (PSGL‐1)‐AF647, FR4‐PE‐Cy7, and PD‐1 (CD279)‐BV421 (BD, eBioscience). Additionally, the live/dead cell dye APC‐Cy7 (BD) was used for cell viability staining and analysis. All antibodies were diluted in PBS before use.

Splenocytes were stimulated with 10 µg/mL purified PR8 (H1N1) or X31 (H3N2) virus for 2 h,, and subsequently incubated with brefeldin A at 37°C for 14 h. The cells were then stained with murine antibodies against phenotypic markers (CD3ε‐BV510, CD4‐FITC, and CD8α‐PerCP‐Cy5.5; BD) and cytokine expression markers (IL‐2‐BV421, IL‐4‐APC, IFN‐γ‐PE‐Cy7, and TNF‐PE; BD). A live/dead cell dye, APC‐Cy7 (BD), was used for viability staining and analysis. Finally, all the samples were analyzed using an LSRFortessa cell analyzer (BD), and the data were processed with FlowJo software (BD). Complete information on the flow cytometry antibodies is provided in Supplementary Data 3.

### 4.11 ELISpot Assays

The production of cytokines in splenocytes was evaluated using ELISpot assays. In brief, 96‐well ELISpot plates (Millipore) precoated with anti‐mouse IFN‐γ, anti‐IL‐4, or anti‐IL‐17A monoclonal antibodies were activated with RPMI 1640 medium supplemented with 10% FBS (Gibco) and 1% PS (Gibco) at room temperature for 30 min after washing. Splenocytes from immunized mice were then added to the plates, followed by stimulation with inactivated PR8 or X31 virus and incubation at 37°C with 5% CO_2_ for 36 h. The cells were subsequently removed, and the plates were washed. Diluted biotin‐conjugated antibodies (Mabtech) were added, and the samples were incubated at 37°C for 2 h. Following another wash, streptavidin–HRP antibodies (Mabtech) were added, and the samples were kept at 37°C for 1 h. Finally, the plates were treated with TMB substrate solution (Mabtech) for 10 min, and the reaction was terminated by washing with deionized water. After drying, the spots were quantified with the AID iSpot reader system. This software calculates the total number of spots per well and provides size and intensity distribution data. The results were exported to Excel for further analysis.

### 4.12 Histological Examination of Mouse Lung Tissues

Mouse lung tissues obtained after challenge were preserved in 10% neutral‐buffered formalin for 24 h, then subjected to a series of graded ethanol treatments (70, 80, 95, and 100%)for dehydration, followed by clearing with xylene and paraffin embedding. For histopathological analysis, the sections were stained using hematoxylin and eosin. In brief, the slides were first immersed in Mayer's hematoxylin for 5 min, followed by rinsing under tap water, differentiated in 1% acid alcohol, and a brief counterstaining with eosin Y for 30 s. After dehydration through a series of graded alcohol and xylene, the sections were mounted with neutral balsam and coverslipped. Observations were made at 40× and 200× magnification using an upright optical microscope (Nikon ECLIPSE CI).

### 4.13 Statistical Analysis

All data analyses were conducted using GraphPad Prism 9 software (GraphPad Software, San Diego, CA). Normality was assessed via the Shapiro–Wilk test (*α* = 0.05), and homogeneity of variance was evaluated with the Brown–Forsythe test. For comparisons among multiple groups, one‐way ANOVA was performed when the data met the parametric assumptions, followed by Tukey's multiple comparisons test for posthoc analysis. For nonnormally distributed data, a nonparametric Kruskal–Wallis test was conducted, followed by Dunn's posthoc test. For mouse survival comparisons, the Mantel–Cox log‐rank test was used. The data are expressed as the means ± standard deviations (SDs), and individual data points are shown in scatter plots. Statistical significance was defined as *p* < 0.05.

To compare T_RM_ and B_RM_ cell production in lung and NALT tissues, radar plots were generated. The production levels of CD8 T_RM_ cells, CD4 T_RM_ cells, and B_RM_ cells are represented by light green, dark green, and blue, respectively. Each axis in the radar plot corresponds to a specific memory cell type, and the distance of each point from the center indicates the standardized relative production level. The standardization was calculated as the mean of five data points in each group divided by the maximum mean (max mean) across all groups.

## Author Contributions

X. Ma and Q. Chen conceived and designed the study, with contributions from Y. Shu, T. Bai, Y. Cai, J. Lu, X. Liu, Z. Yang, X. Han, L. Wang, Y. Shi, and Y. Zhang. X. Ma, Q. Chen, Y. Cai, C. Chen, J. Lu, L. Wang, R. Ma, and W. Pang conducted the experiments. X. Ma, Q. Chen, L. Xin, and Y. Chen analyzed and interpreted the results. X. Ma, Q. Chen, and Y. Cai wrote the original manuscript, which was revised and edited by T. Bai and Y. Shu. All authors have read and approved the final manuscript.

## Funding

This project was supported by the Chinese Academy of Medical Sciences Innovation Fund for Medical Sciences [2022‐I2M‐1‐021], the Beijing Natural Science Foundation [L242036], the Chinese Academy of Medical Sciences Innovation Fund for Medical Sciences [2023‐I2M‐2‐001], and the Sanming Project for Medicine in Shenzhen [SZSM202103008].

## Conflicts of Interest

All authors declare no conflicts of interest.

## Ethics Statement

This study was conducted in strict accordance with the guidelines outlined in the Guide for the Care and Use of Laboratory Animals, which was issued by the Ministry of Science and Technology of the People's Republic of China. All experimental procedures were approved by the China National Institute for Food and Drug Control Laboratory Animal Welfare and Ethics Committee (approval number: 2024(B)002). Under strict ethical supervision, the experiments were carried out in accordance with established safety standards. Experiments involving live viruses and animals were conducted in negative‐pressure isolators with HEPA filters in the animal biosafety level 2 (ABSL2) laboratory at the National Institute for Food and Drug Control Laboratory Animal Center and the biosafety level 2 (BSL2) laboratory at the National Institute of Pathogen Biology.

## Supporting information




**Figure S1**: Comparisons of the HA and NA sequences of representative H1N1 strains from 1970 to the present with those of 09‐22H1m and 09‐22N1m, respectively.
**Figure S2**: Histological analysis of tissues from immunized mice. Observations were made at 40× (scale bar: 1000 µm) and 200× (scale bar: 200 µm) magnification.
**Figure S3**: Representative flow cytometry results for CD8+ IL‐2+, CD8+ IL‐4+, CD8+ IFN‐γ+, CD8+ TNF‐α+, CD4+ IL‐2+, CD4+ IL‐4+, CD4+ IFN‐γ+, and CD4+ TNF‐α+ cells after stimulation with purified PR8 or X31 antigen.
**Figure S4**: Flow cytometry gating strategies for CD8+ IL‐2+, CD8+ IL‐4+, CD8+ IFN‐γ+, CD8+ TNF‐α+, CD4+ IL‐2+, CD4+ IL‐4+, CD4+ IFN‐γ+, and CD4+ TNF‐α+ cells after stimulation with purified PR8 or X31 antigen.
**Figure S5**: Representative flow cytometry results for CD69+ CD8+ T, CD69+ CD103+ CD8+ T, CD69+ CD4+ T, TRH, Trm1, IgM+ memory B and ISW memory B cells in the lungs, and CD69+ CD8+ T, CD69+ CD103+ CD8+ T, CD69+ CD4+ T, Tfh, Th1, IgM+ memory B and ISW memory B cells in the NALT.
**Figure S6**: Flow cytometry gating strategies for CD69+ CD8+ T, CD69+ CD103+ CD8+ T, CD69+ CD4+ T, TRH, Trm1, IgM+ memory B, and ISW memory B cells in the lungs, and CD69+ CD8+ T, CD69+ CD103+ CD8+ T, CD69+ CD4+ T, Tfh, Th1, IgM+ memory B and ISW memory B cells in the NALT.
**Figure S7**: Histological analysis of lung tissue from an A/Puerto Rico/8/34 (PR8)‐infected mouse. Observations were made at 40× (scale bar: 1000 µm) and 200× (scale bar: 200 µm) magnification.
**Figure S8**: Histological analysis of lung tissue from an A/Hunan/42443/2015 (swH1N1)‐infected mouse. Observations were made at 40× (scale bar: 1000 µm) and 200× (scale bar: 200 µm) magnification.
**Figure S9**: Histological analysis of lung tissue from an A/Victoria/4897/2022 (Vic22)‐infected mouse. Observations were made at 40× (scale bar: 1000 µm) and 200× (scale bar: 200 µm) magnification.
**Figure S10**: Histological analysis of lung tissue from a mouse infected with heterologous A/Aichi/2/1968 (X31, H3N2). Observations were made at 40× (scale bar: 1000 µm) and 200× (scale bar: 200 µm) magnification.
**Supplementary Data 1**: mH1 and mN1 mosaic nucleotide sequences.
**Supplementary Data 2**: Information on the influenza strains and their corresponding WHO‐recommended years.
**Supplementary Data 3**: Information on flow cytometry antibodies.

## Data Availability

All data generated in this study are available within the article and its supplementary information. The influenza sequences used for immunogen design are publicly accessible via the Influenza Virus Database (NCBI and GISAID). The mosaic vaccine designer algorithm and related tools (Epicover, Posicover) are available at LANL HIV Database (https://www.hiv.lanl.gov/content/sequence/MOSAIC/makeVaccine.html). All other data are available from the corresponding author upon reasonable request.
