# Supplementary material for: Intranasal Mosaic H1N1 Live Attenuated Influenza Vaccine Elicits Broad Cross‐Reactive Immunity and Protection Against Group 1 and 2 Influenza A Viruses
Source: MedComm (2020). 2025 Dec 15;6(12):e70557. doi: 10.1002/mco2.70557 (PMC12706173; doi:10.1002/mco2.70557)
Supplement: Supplementary file 1 — Figure S1: Comparisons of the HA and NA sequences of representative H1N1 strains from 1970 to the present with those of 09‐22H1m and 09‐22N1m, respectively. Figure S2: Histological analysis of tissues from immunized mice. Observations were made at 40× (scale bar: 1000 µm) and 200× (scale bar: 200 µm) magnification. Figure S3: Representative flow cytometry results for CD8+ IL‐2+, CD8+ IL‐4+, CD8+ IFN‐γ+, CD8+ TNF‐α+, CD4+ IL‐2+, CD4+ IL‐4+, CD4+ IFN‐γ+, and CD4+ TNF‐α+ cells after stimulation with purified PR8 or X31 antigen. Figure S4: Flow cytometry gating strategies for CD8+ IL‐2+, CD8+ IL‐4+, CD8+ IFN‐γ+, CD8+ TNF‐α+, CD4+ IL‐2+, CD4+ IL‐4+, CD4+ IFN‐γ+, and CD4+ TNF‐α+ cells after stimulation with purified PR8 or X31 antigen. Figure S5: Representative flow cytometry results for CD69+ CD8+ T, CD69+ CD103+ CD8+ T, CD69+ CD4+ T, TRH, Trm1, IgM+ memory B and ISW memory B cells in the lungs, and CD69+ CD8+ T, CD69+ CD103+ CD8+ T, CD69+ CD4+ T, Tfh, Th1, IgM+ memory B and ISW memory B cells in the NALT. Figure S6: Flow cytometry gating strategies for CD69+ CD8+ T, CD69+ CD103+ CD8+ T, CD69+ CD4+ T, TRH, Trm1, IgM+ memory B, and ISW memory B cells in the lungs, and CD69+ CD8+ T, CD69+ CD103+ CD8+ T, CD69+ CD4+ T, Tfh, Th1, IgM+ memory B and ISW memory B cells in the NALT. Figure S7: Histological analysis of lung tissue from an A/Puerto Rico/8/34 (PR8)‐infected mouse. Observations were made at 40× (scale bar: 1000 µm) and 200× (scale bar: 200 µm) magnification. Figure S8: Histological analysis of lung tissue from an A/Hunan/42443/2015 (swH1N1)‐infected mouse. Observations were made at 40× (scale bar: 1000 µm) and 200× (scale bar: 200 µm) magnification. Figure S9: Histological analysis of lung tissue from an A/Victoria/4897/2022 (Vic22)‐infected mouse. Observations were made at 40× (scale bar: 1000 µm) and 200× (scale bar: 200 µm) magnification. Figure S10: Histological analysis of lung tissue from a mouse infected with heterologous A/Aichi/2/1968 (X31, H3N2). Observatio [file MCO2-6-e70557-s001.pdf]

Supplemental Information

Intranasal Mosaic H1N1 Live Attenuated Influenza Vaccine Elicits Broad Cross-Reactive Immunity and Protection Against Group 1 and 2 Influenza A Viruses

Ximeng Ma [1#], Qi Chen [1#], Yukun Cai [3], Chen Chen [1], Jian Lu[4], Zhuolin Yang [2], Xue Han [1], Liangliang Wang [4], Xuejie Liu [2,5], Yuhang Shi [1], Yuhang Zhang [1], Li Xin[6], Yihao Chen [1], Run Ma [1], Wantong Pang [1], Tian Bai [1*], Yuelong Shu [1,2*]

1 Key Laboratory of Pathogen Infection Prevention and Control (Peking Union Medical College, Ministry of Education), State Key Laboratory of Respiratory Health and Multimorbidity, National Institute of Pathogen Biology of Chinese Academy of Medical Science (CAMS)/Peking Union Medical College (PUMC), Beijing 100730, China

2 School of Public Health (Shenzhen), Shenzhen Campus of Sun Yat-sen University, Shenzhen 518107, China

3 Department of Comprehensive Plastic Surgery, Plastic Surgery Hospital, Chinese Academy of Medical Sciences and Peking Union Medical College, Beijing 100144, China.

4 National Institute for Viral Disease Control and Prevention, Collaborative Innovation Center for Diagnosis and Treatment of Infectious Diseases, China CDC, 155 Changbai Road, Beijing, 102206, P. R. China

5 Division of HIV/AIDS and Sex-transmitted Virus Vaccines, Institute for Biological Product Control, National Institutes for Food and Drug Control (NIFDC), Beijing, People's Republic of China.

6 Section of Infectious Diseases, Department of Internal Medicine, Yale University School of Medicine, New Haven, CT, USA.

Collaborating Center for Standardization and Evaluation of Biologicals, No. 31 Huatuo Street, Daxing District, Beijing 102629, China

# Contributed equally

*Correspondence: shuylong@mail.sysu.edu.cn (Y.S.), baitian@ipbcams.ac.cn (T.B.)

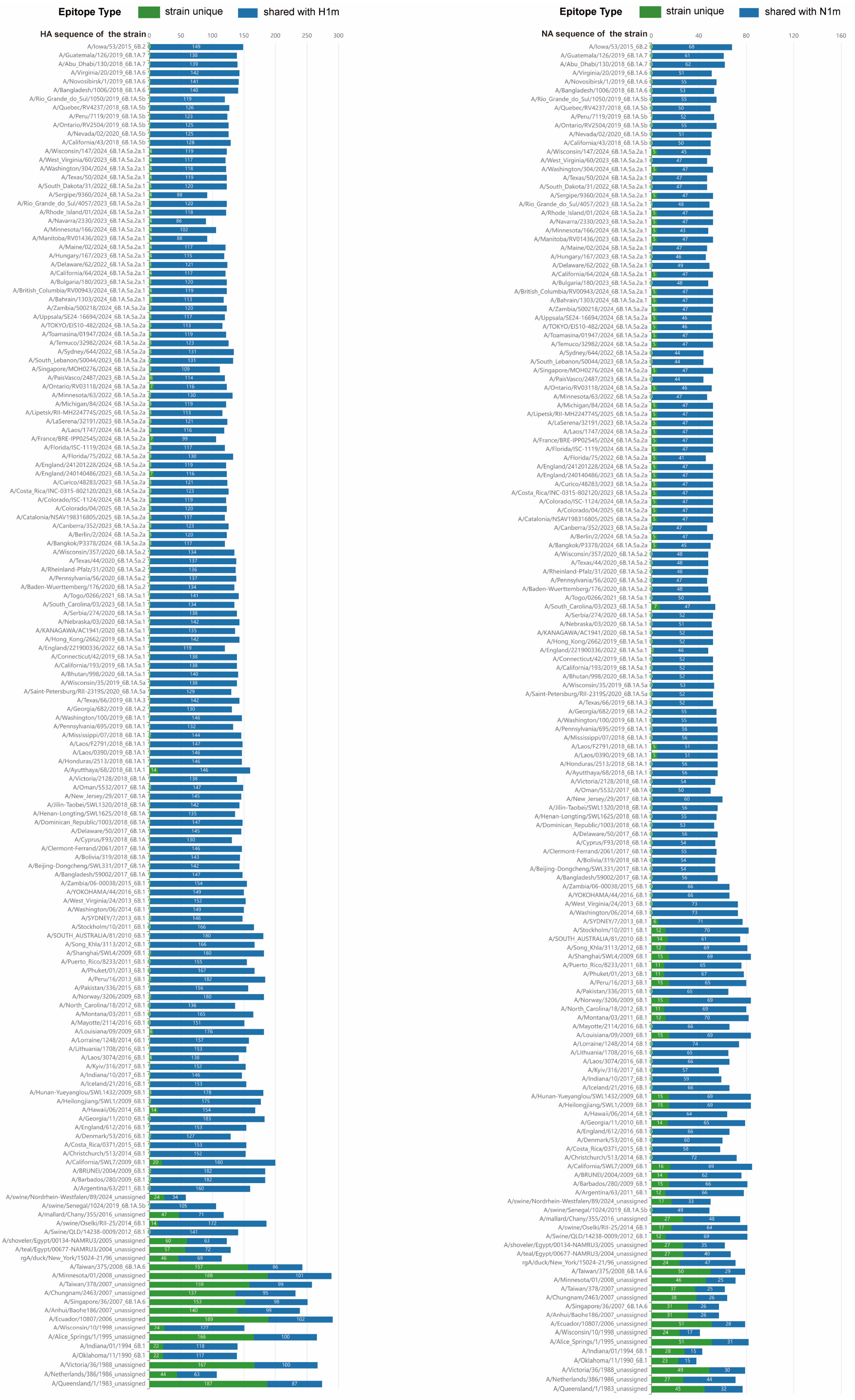

Figure S1. Comparisons of the HA and NA sequences of representative H1N1 strains from 1970 to the present with those of 09-22H1m and 09-22N1m, respectively.

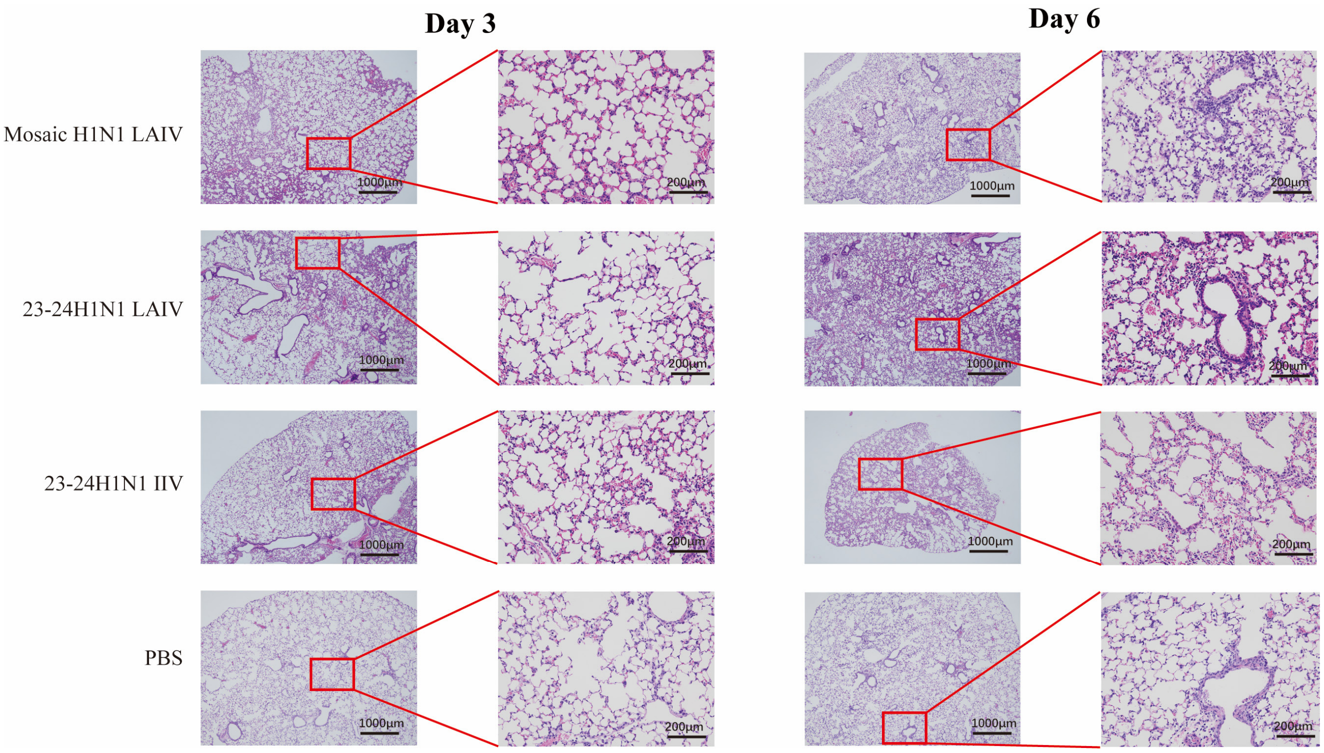

Figure S2. Histological analysis of tissues from immunized mice. Observations were made at 40× (scale bar: 1000 μm) and 200× (scale bar: 200 μm) magnification.

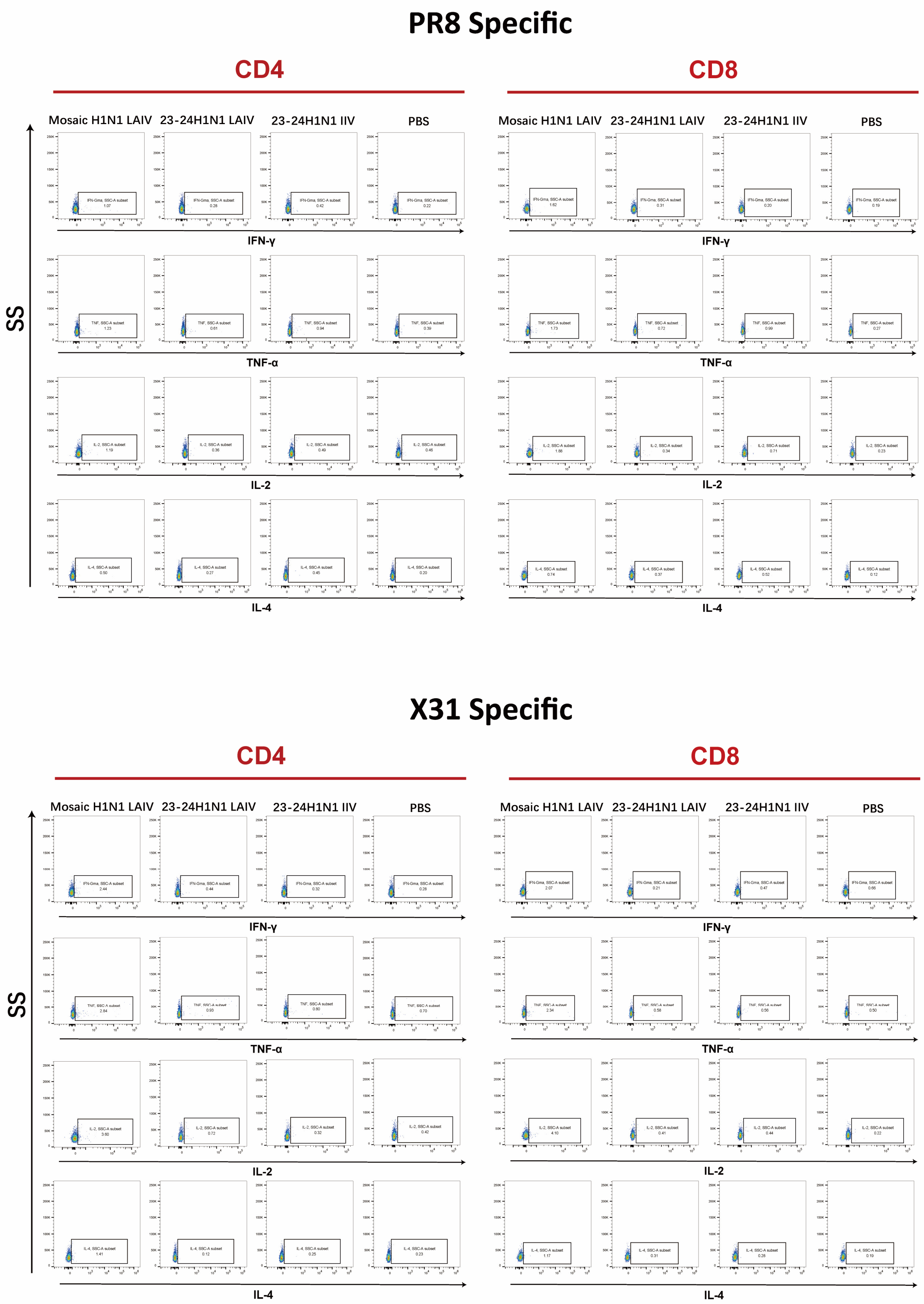

Figure S3. Representative flow cytometry results for CD8⁺ IL-2⁺, CD8⁺ IL-4⁺, CD8⁺ IFN-γ⁺, CD8⁺ TNF-α⁺, CD4⁺ IL-2⁺, CD4⁺ IL-4⁺, CD4⁺ IFN-γ⁺, and CD4⁺ TNF-α⁺ cells after stimulation with purified PR8 or X31 antigen.

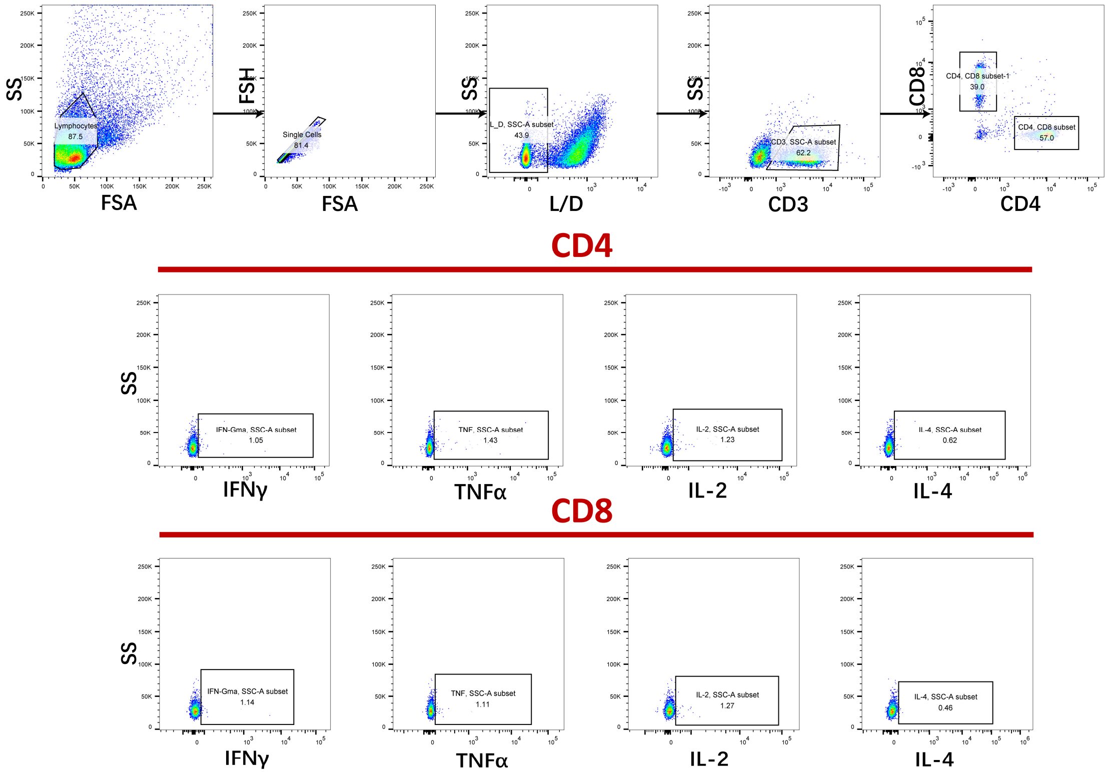

Figure S4. Flow cytometry gating strategies for CD8⁺ IL-2⁺, CD8⁺ IL-4⁺, CD8⁺ IFN-γ⁺, CD8⁺ TNF-α⁺, CD4⁺ IL-2⁺, CD4⁺ IL-4⁺, CD4⁺ IFN-γ⁺, and CD4⁺ TNF-α⁺ cells after stimulation with purified PR8 or X31 antigen.

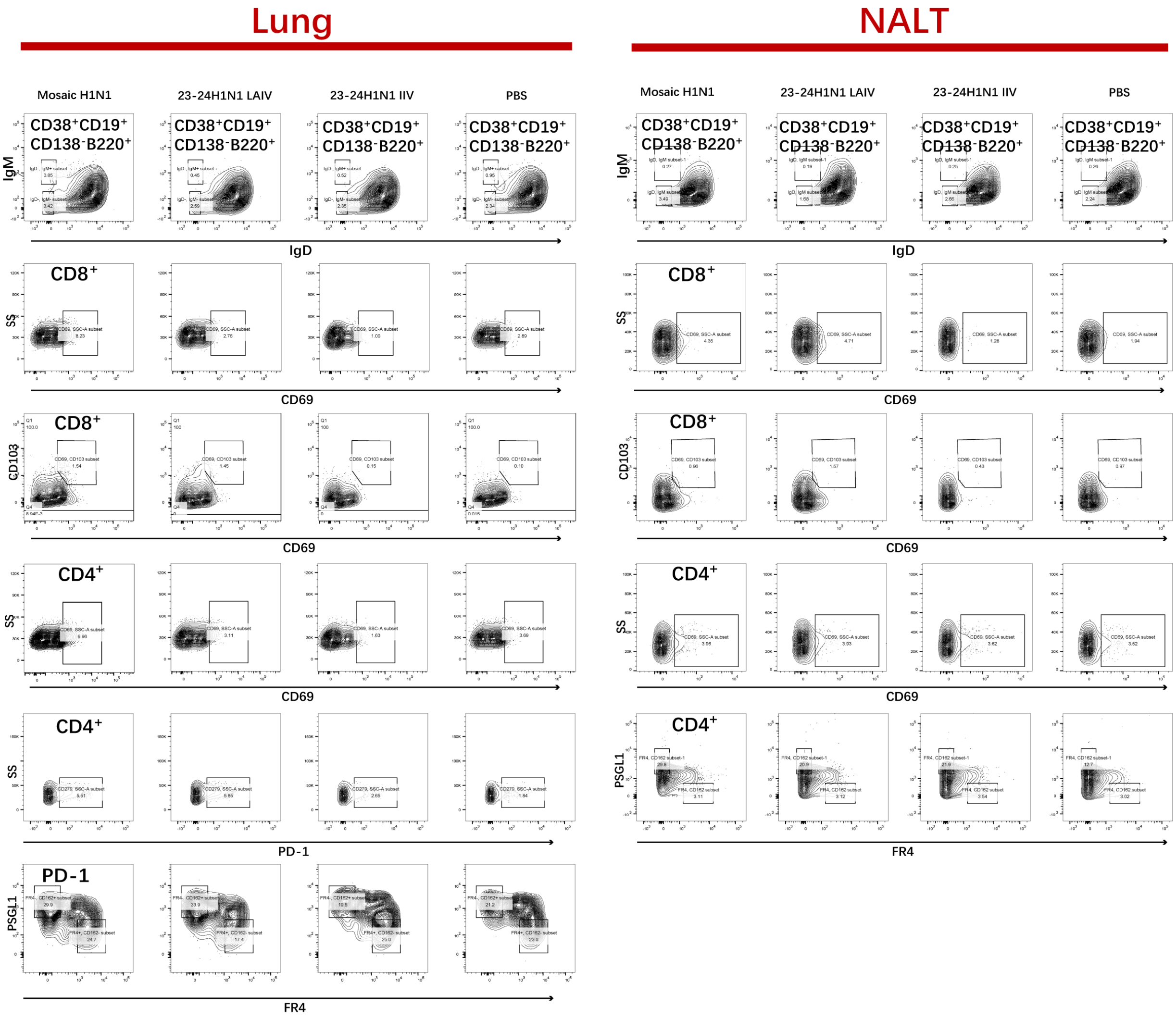

Figure S5. Representative flow cytometry results for CD69⁺ CD8⁺ T, CD69⁺ CD103⁺ CD8⁺ T, CD69⁺ CD4⁺ T, T_{RH}, T_{rm1}, IgM⁺ memory B and ISW memory B cells in the lungs, and CD69⁺ CD8⁺ T, CD69⁺ CD103⁺ CD8⁺ T, CD69⁺ CD4⁺ T, T_{fh}, T_{h1}, IgM⁺ memory B and ISW memory B cells in the NALT.

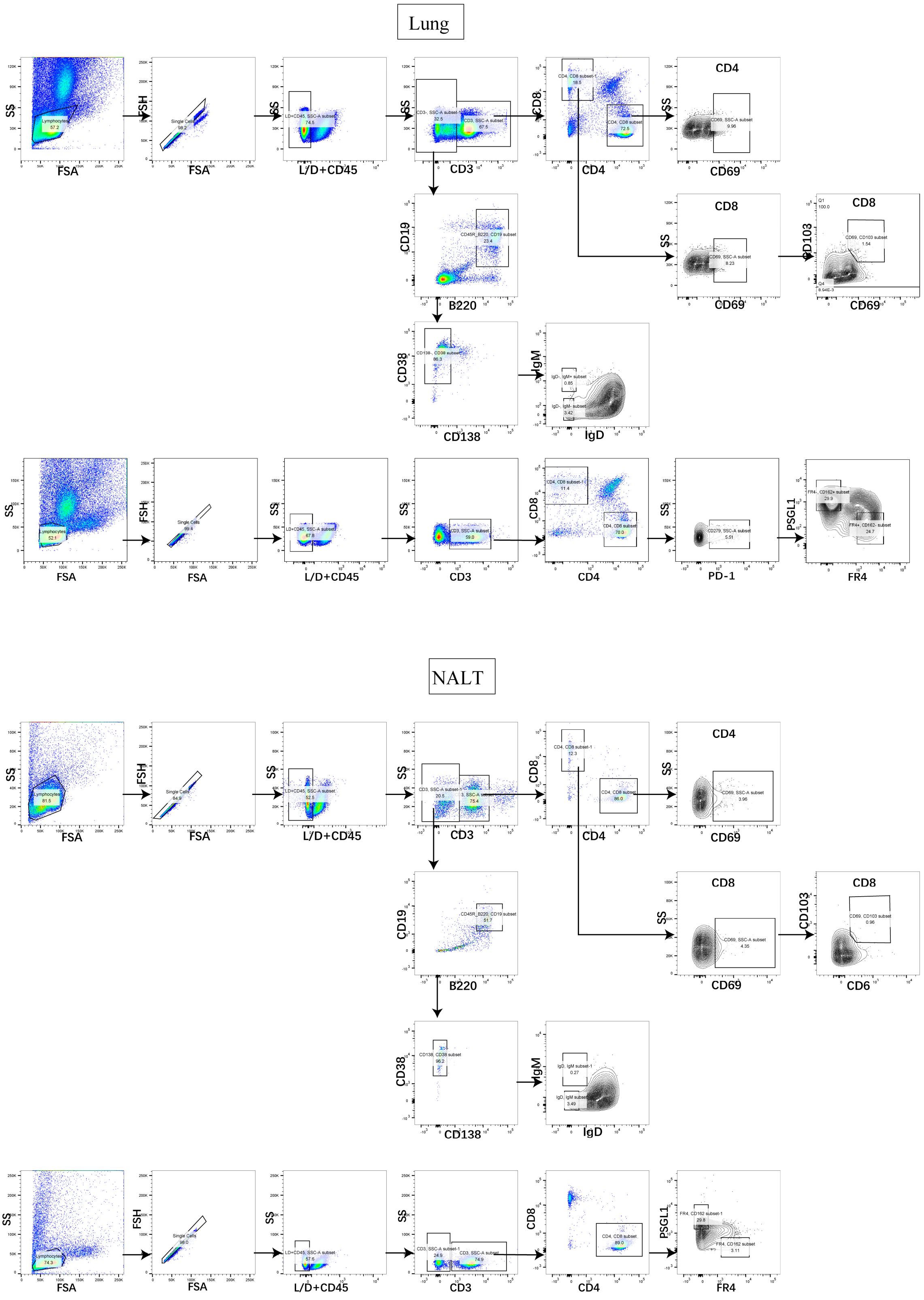

Figure S6. Flow cytometry gating strategies for CD69$^+$ CD8$^+$ T, CD69$^+$ CD103$^+$ CD8$^+$ T, CD69$^+$ CD4$^+$ T, T_{RH}, T_{rm1}, IgM$^+$ memory B, and ISW memory B cells in the lungs, and CD69$^+$ CD8$^+$ T, CD69$^+$ CD103$^+$ CD8$^+$ T, CD69$^+$ CD4$^+$ T, T_{fh}, T_{h1}, IgM$^+$ memory B and ISW memory B cells in the NALT.

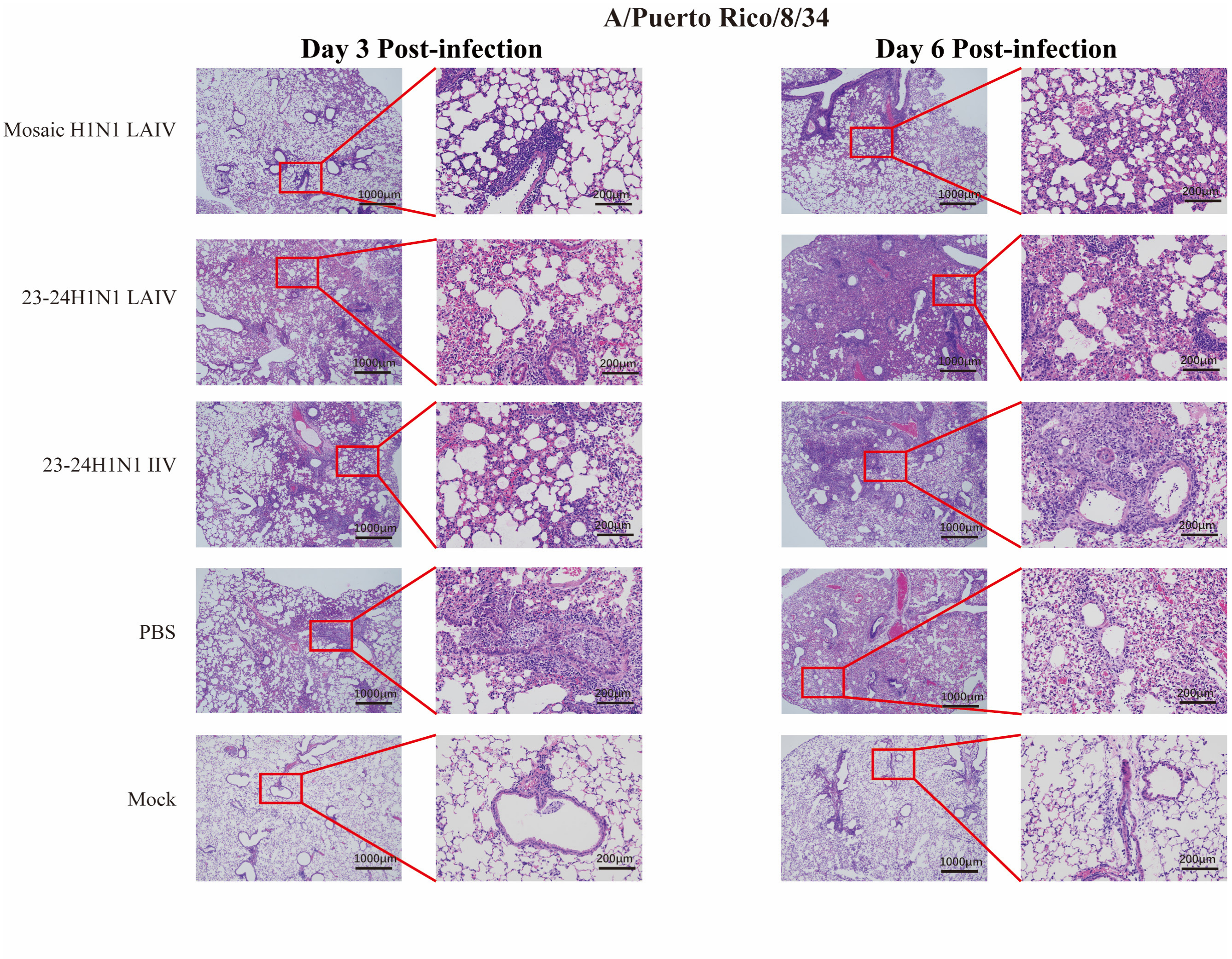

Figure S7. Histological analysis of lung tissue from an A/Puerto Rico/8/34 (PR8)-infected mouse. Observations were made at 40× (scale bar: 1000 μm) and 200× (scale bar: 200 μm) magnification.

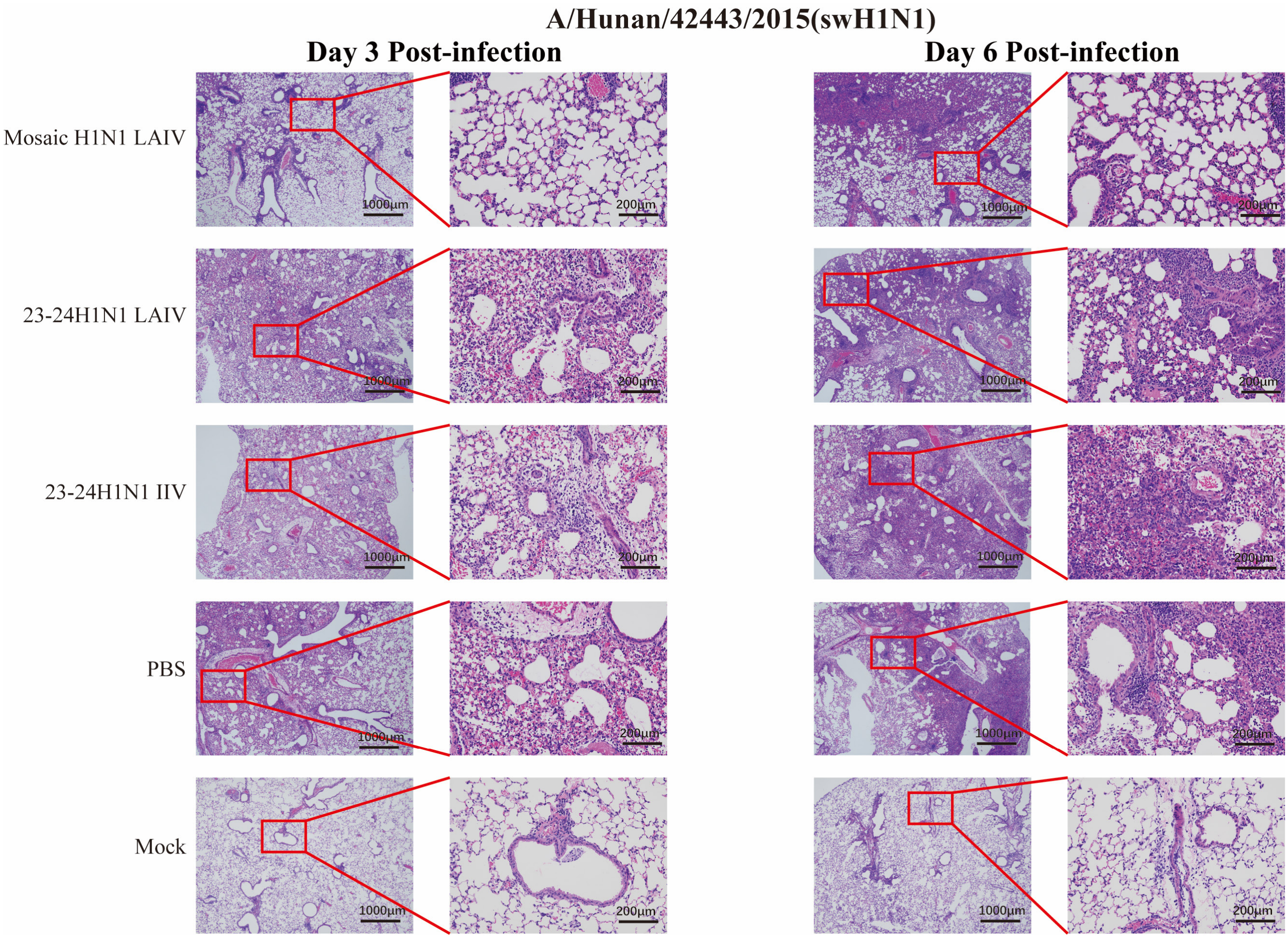

Figure S8. Histological analysis of lung tissue from an A/Hunan/42443/2015 (swH1N1)-infected mouse. Observations were made at 40× (scale bar: 1000 μm) and 200× (scale bar: 200 μm) magnification.

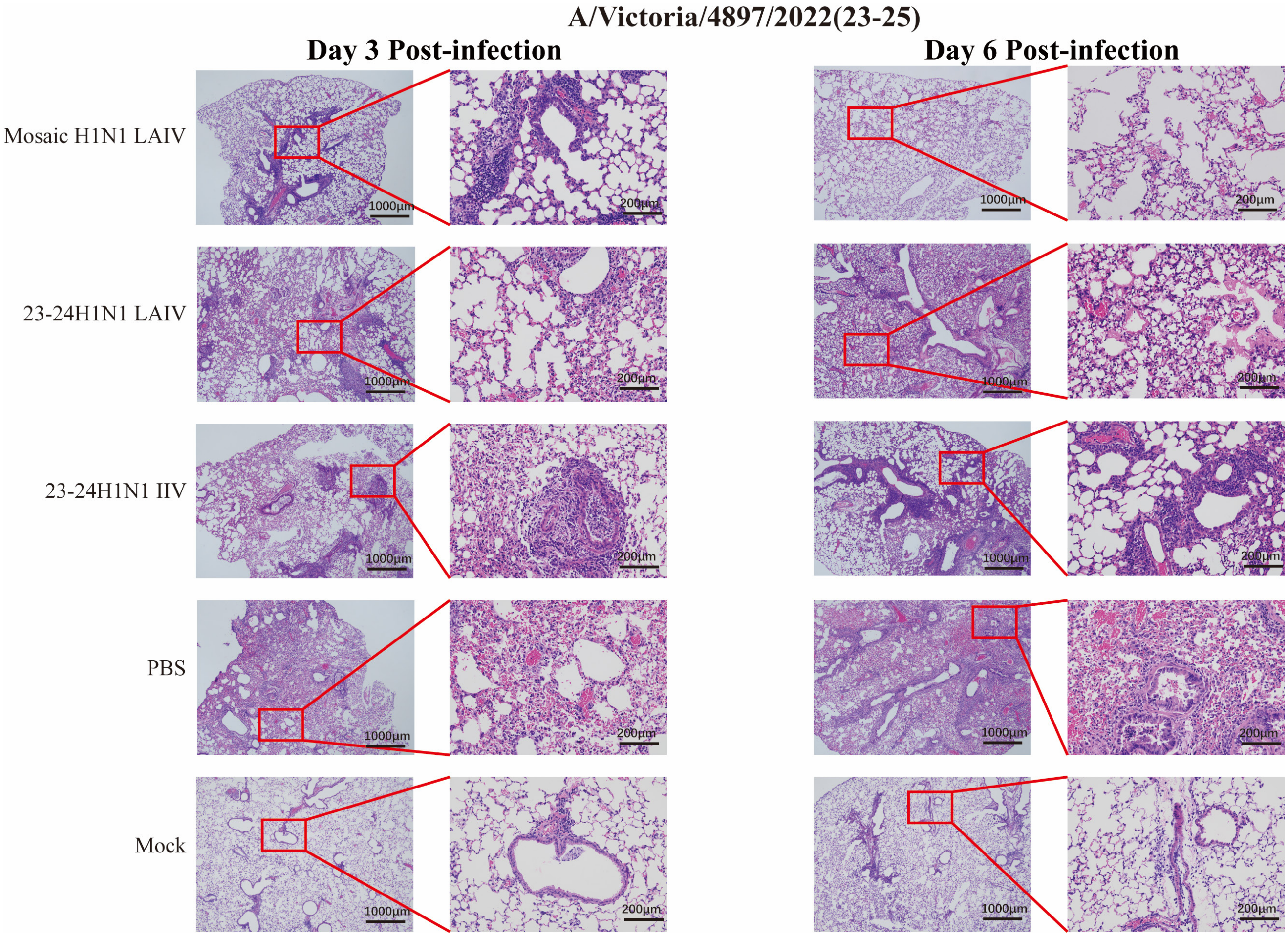

Figure S9. Histological analysis of lung tissue from an A/Victoria/4897/2022 (Vic22)-infected mouse. Observations were made at 40× (scale bar: 1000 μm) and 200× (scale bar: 200 μm) magnification.

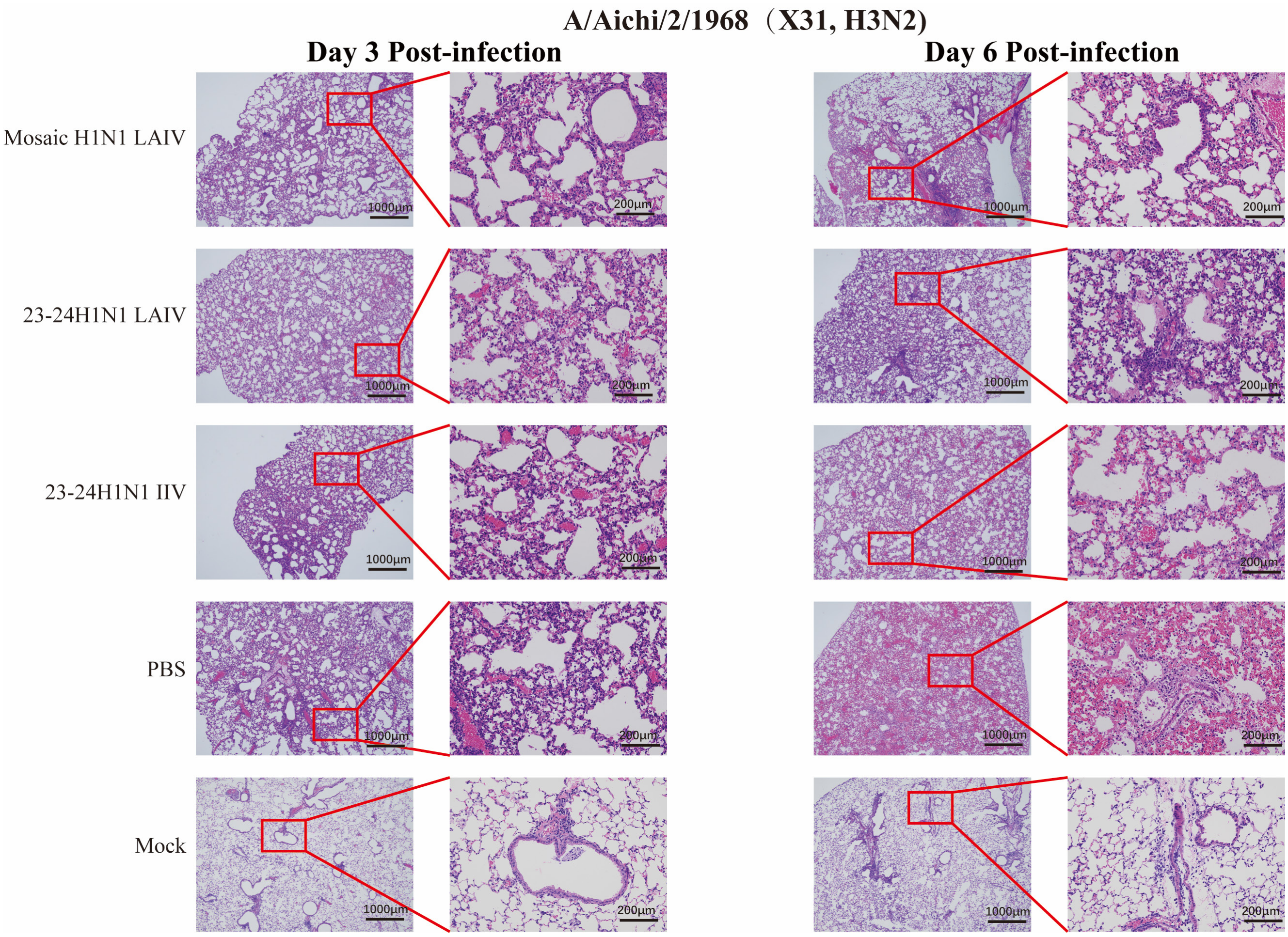

Figure S10. Histological analysis of lung tissue from a mouse infected with heterologous A/Aichi/2/1968 (X31, H3N2). Observations were made at 40× (scale bar: 1000 µm) and 200× (scale bar: 200 µm) magnification.

>mHA
AGCAAAAGCAGGGGAAAACAAAAGCAACAAAAATGAAGGCAATACTAGTAGTTCTGCTATATACATTTGCAACCGCAAAT
GCAGACACATTATGTATAGGTTATCATGCGAACAATTCAACAGACACTGTAGACACAGTACTAGAAAAGAATGTAACAGTA
ACACACTCTGTTAACCTTCTAGAAGACAAGCATAACGGGAAACTATGCAAACTAAGAGGGGTAGCCCCATTGCATTTGGG
TAAATGTAACATTGCTGGCTGGATCCTGGGAAATCCAGAGTGTGAATCACTCTCCACAGCAAGCTCATGGTCCTACATTGT
GGAAACATCTAGTTCAGACAATGGAACGTGTTACCCAGGAGATTTCATCGATTATGAGGAGCTAAGAGAGCAATTGAGCT
CAGTGTCATCATTTGAAAGGTTTGAGATATTCCCCAAGACAAGTTCATGGCCCAATCATGACTCGAACAAAGGTGTAACGG
CAGCATGTCCTCATGCTGGAGCAAAAAGCTTCTACAAAAATTTAATATGGCTAGTTAAAAAAGGAAATTCATACCCAAAGC
TCAGCAAATCCTACATTAATGATAAAGGGAAAGAAGTCCTCGTGCTATGGGGCATTCACCATCCATCTACTAGTGCTGACC
AACAAAGTCTCTATCAGAATGCAGATGCATATGTTTTTGTGGGGACATCAAGATACAGCAAGAAGTTCAAGCCGGAAATA
GCAATAAGACCCAAAGTGAGGGATCAAGAAGGGAGAATGAACTATTACTGGACACTAGTAGAGCCGGGAGACAAAATAA
CATTCGAAGCAACTGGAAATCTAGTGGTACCGAGATATGCATTCGCAATGGAAAGAAATGCTGGATCTGGTATTATCATTT
CAGATACACCAGTCCACGATTGCAATACAACTTGTCAGACACCCAAGGGTGCTATAAACACCAGCCTCCCATTTCAGAATA
TACATCCGATCACAATTGGAAAATGTCCAAAATATGTAAAAAGCACAAAATTGAGACTGGCCACAGGATTGAGGAATGTC
CCGTCTATTCAATCTAGAGGCCTATTTGGGGCCATTGCCGGTTTCATTGAAGGGGGGTGGACAGGGATGGTAGATGGATG
GTACGGTTATCACCATCAAAATGAGCAGGGGTCAGGATATGCAGCCGACCTGAAGAGCACACAGAATGCCATTGACAAA
ATTACTAACAAAGTAAATTCTGTTATTGAAAAGATGAATACACAGTTCACAGCAGTAGGTAAAGAGTTCAACCACCTGGAA
AAAAGAATAGAGAATTTAAATAAAAAAGTTGATGATGGTTTCCTGGACATTTGGACTTACAATGCCGAACTGTTGGTTCTAT
TGGAAAATGAAAGAACTTTGGACTACCACGATTCAAATGTGAAGAACTTATATGAAAAGGTAAGAAGCCAGTTAAAAAAC
AATGCCAAGGAAATTGGAAACGGCTGCTTTGAATTTTACCACAAATGCGATAACACGTGCATGGAAAGTGTCAAAAATGG
GACTTATGACTACCCAAAATACTCAGAGGAAGCAAAATTAAACAGAGAAGAAATAGATGGGGTAAAGCTGGAATCAACA
AGGATTTACCAGATTTTGGCGATCTATTCAACTGTCGCCAGTTCATTGGTACTGGTAGTCTCCCTGGGGGCAATCAGTTTCT
GGATGTGCTCTAATGGGTCTCTACAGTGTAGAATATGTATTTAACATTAGGATTTCAGAAGCATGAGAAAAACACCCTTGTT
TCTACT

>mNA
AGCAAAAGCAGGAGTTTAAAATGAATCCAAACCAAAAGATAATAACCATTGGTTCGGTCTGTATGACAATTGGAATGGCTA
ACTTAATATTACAAATTGGAAACATAATCTCAATATGGGTTAGCCACTCAATTCAAATTGGGAATCAAAGTCAGATTGAAAC
ATGCAATCAAAGCGTCATTACTTATGAAAACAACACTTGGGTAAACCAGACATATGTTAACATCAGCAACACCAACTTTGC
TGCTGGACAGTCAGTGGTTTCCGTGAAATTAGCGGGCAATTCCTCTCTCTGCCCTGTTAGTGGATGGGCTATATACAGTAAA
GACAACAGTGTAAGAATCGGTTCCAAGGGGGATGTGTTTGTCATAAGGGAACCATTCATATCATGCTCTCCCTTGGAATGC
AGAACCTTCTTCTTGACTCAAGGGGCCTTGCTAAATGACAAACATTCCAATGGAACCATTAAAGACAGGAGCCCATATCGA
ACCCTAATGAGCTGTCCTATTGGTGAAGTTCCCTCTCCATACAACTCAAGATTTGAGTCAGTCGCTTGGTCAGCAAGTGCTT
GTCATGATGGCATCAATTGGCTAACAATTGGAATTTCTGGCCCAGACAGTGGGGCAGTGGCTGTGTTAAAGTACAATGGCA
TAATAACAGACACTATCAAGAGTTGGAGGAACAATATATTGAGAACACAAGAGTCTGAATGTGCATGTGTAAATGGTTCTT
GCTTTACCATAATGACCGATGGACCAAGTGATGGACAGGCCTCATACAAGATCTTCAGAATAGAAAAGGGAAAGATAGTC
AAATCAGTCGAAATGAATGCCCCTAATTATCACTATGAGGAATGCTCCTGTTATCCTGATTCTAGTGAAATCACATGTGTGT
GCAGGGATAACTGGCATGGCTCGAATCGACCGTGGGTGTCTTTCAACCAGAATCTGGAATATCAGATAGGATACATATGC
AGTGGGGTTTTCGGAGACAATCCACGCCCTAATGATAAGACAGGCAGTTGTGGTCCAGTATCGTCTAATGGAGCAAATGG
AGTAAAAGGATTTTCATTCAAATACGGCAATGGTGTTTGGATAGGGAGAACTAAAAGCATTAGTTCAAGAAAAGGTTTTGA
GATGATTTGGGATCCGAATGGATGGACTGGGACTGACAATAACTTCTCAATAAAGCAAGATATCGTAGGAATAAATGAGT
GGTCAGGATATAGCGGGAGTTTTGTTCAGCATCCAGAACTAACAGGGCTGGATTGTATAAGACCTTGCTTTTGGGTTGAAC
TAATCAGAGGGCGACCCGAAGAGAACACAATCTGGACTAGCGGGAGCAGCATATCCTTTTGTGGTGTAAACAGTGACACT
GTGGGTTGGTCTTGGCCAGACGGTGCTGAGTTGCCATTTACCATTGACAAGTAATTTGTTCAAAAAACTCCTTGTTTCTACT

Supplementary Data 1. mH1 and mN1 mosaic nucleotide sequences.

Strains (H1N1)	Indicators	Details
**A/Victoria/4897/2022 (Vic22)**	ELISA、HAI、MN	2023-2025<br>WHO recommended
**A/Victoria/2570/2019 (Vic19)**	ELISA、HAI、MN	2021-2023<br>WHO recommended
**A/Guangdong-Maonan/SWL1536/2019 (Mao19)**	ELISA、HAI、MN	2020-2021<br>WHO recommended
**A/Brisbane/02/2018 (Bris18)**	ELISA、HAI、MN	2019-2020<br>WHO recommended
**A/Michigan/45/2015 (Mich15)**	ELISA、HAI、MN	2017-2019<br>WHO recommended
**A/California/7/2009 (CA07)**	ELISA、HAI、MN	2013-2017<br>WHO recommended
**A/California/04/2009 (CA04)**	HAI、MN	Pandemic 2009
**A/Hunan/42443/2015 (swH1N1)**	ELISA、HAI、MN	Swine influenza
**A/Puerto Rico/8/1934 (PR8)**	ELISA、HAI、MN	Ancestral<br>H1N1 strain
**A/Hongkong/2671/2019 (H3N2, HK19)**	ELISA、HAI	H3N2 strain
**A/Darwin/9/2021 (H3N2, Darwin21)**	ELISA、HAI	H3N2 strain
**A/Aichi/2/1968 (H3N2, X31)**	ELISA、HAI	Ancestral<br>H3N2 strain
**A/Hongkong/1073/99 (H9N2, HK99)**	ELISA、HAI	H9N2 strain
**A/Cambodia/R0405050/2007<br>(H5N1, Cam07)**	ELISA、HAI	H5N1 strain

Supplementary Data 2. Information on the influenza strains and their corresponding WHO-recommended years.

Antibody	Brand	Cat NO.
**Pan Influenza A Nucleoprotein Antibody, Rabbit MAb**	Sino Biological	40208-R010
**Goat Anti-Rabbit IgG Secondary Antibody (HRP)**	Sino Biological	SSA004
**Anti-Mouse IgA (α-chain specific) antibody produced in goat**	Sigma–Aldrich	M8769-1MG
**goat anti-mouse IgA alpha chain (HRP)**	Abcam	AB97235
**APC-Cy7 FVS780**	BD	565388
**CD45-APC-Cy7**	(BD)	557659
**CD3ε-BV605**	BD	563004
**CD4-RB780**	BD	570257
**CD8α-BV510**	BD	563068
**CD69-APC**	BD	560689
**CD103-BV786**	BD	564322
**CD45R/B220-FITC**	BD	553079
**CD38-BV421**	BD	562768
**CD138-BV650**	BD	564068
**CD19-PE**	BD	557399
**IgD-BV711**	BD	564275
**IgM-PE-Cy7**	BD	552867
**CD3ε-BV605**	BD	563004
**CD4-RB780**	BD	570257
**CD162(PSGL-1)-AF647**	BD	562806
**FR4-PE-Cy7**	ebioscience	25-5445-82
**PD-1(CD279)-BV421**	BD	562584
**CD3ε-BV510**	BD	563024
**CD4-FITC**	BD	553046
**CD8α-PerCP-Cy5.5**	BD	551162
**IL-2-BV421**	BD	562969
**IL-4-APC**	BD	554436
**IFN-γ-PE-Cy7**	BD	557649
**TNF-PE**	BD	554419

Supplementary Data 3. Information on flow cytometry antibodies**.**
